# Immunopeptidomic analyses of colorectal cancers with and without microsatellite instability

**DOI:** 10.1016/j.mcpro.2022.100228

**Published:** 2022-04-01

**Authors:** Jenna Cleyle, Marie-Pierre Hardy, Robin Minati, Mathieu Courcelles, Chantal Durette, Joel Lanoix, Jean-Philippe Laverdure, Krystel Vincent, Claude Perreault, Pierre Thibault

**Affiliations:** 1 Institute for Research in Immunology and Cancer, Université de Montréal, Montreal, Quebec, Canada; 2 Molecular Biology Program, Université de Montréal, Montreal, Quebec, Canada; 3 Department of Medicine, Université de Montréal, Montreal, Quebec, Canada; 4 Department of Chemistry, Université de Montréal, Montreal, Quebec, Canada

**Keywords:** colorectal cancer, tumor-specific antigen, cancer immunotherapy, immunopeptidomics, mass spectrometry, aeTSA, aberrantly expressed tumor-specific antigen, AGC, automatic gain control, AML, acute myeloid leukemia, ATCC, American Type Culture Collection, CEA, carcinoembryonic antigen, COAD, colon adenocarcinoma, CRC, colorectal cancer, CTA, cancer testis antigen, DEG, differentially expressed gene, ERE, endogenous retroviral element, FA, formic acid, FBS, fetal bovine serum, FDR, false discovery rate, GO, gene ontology, GTEx, Genotype Tissue Expression project, HLA, human leukocyte antigen, ICI, immune checkpoint inhibition, IEDB, Immune Epitope Database, INDEL, insertion/deletion, KPHM, kmers-per-hundred-million, LC-MS/MS, liquid chromatography tandem mass spectrometry, lncRNA, long noncoding RNA, MAP, MHC I-associated peptide, MCS, MAP-coding sequence, MHC, major histocompatibility complex, MS, mass spectrometry, MSI, microsatellite instability, MSS, microsatellite stable, mTEC, medullary thymic epithelial cell, mTSA, mutated tumor-specific antigen, NAT, normal adjacent tissue, PSM, peptide spectrum match, RPHM, reads-per-hundred-million, SNV, single nucleotide variant, SPS-MS3, synchronous precursor selection MS3, TAA, tumor associated antigen, TCGA, The Cancer Genome Atlas, TCR, T cell receptor, TEC, Thymic epithelial cell, TMT, tandem mass tag, TPM, transcripts per million, TSA, Tumor-specific antigen

## Abstract

Colorectal cancer is the second leading cause of cancer death worldwide, and the incidence of this disease is expected to increase as global socioeconomic changes occur. Immune checkpoint inhibition therapy is effective in treating a minority of colorectal cancer tumors; however, microsatellite stable tumors do not respond well to this treatment. Emerging cancer immunotherapeutic strategies aim to activate a cytotoxic T cell response against tumor-specific antigens, presented exclusively at the cell surface of cancer cells. These antigens are rare and are most effectively identified with a mass spectrometry–based approach, which allows the direct sampling and sequencing of these peptides. Although the few tumor-specific antigens identified to date are derived from coding regions of the genome, recent findings indicate that a large proportion of tumor-specific antigens originate from allegedly noncoding regions. Here, we employed a novel proteogenomic approach to identify tumor antigens in a collection of colorectal cancer–derived cell lines and biopsy samples consisting of matched tumor and normal adjacent tissue. The generation of personalized cancer databases paired with mass spectrometry analyses permitted the identification of more than 30,000 unique MHC I–associated peptides. We identified 19 tumor-specific antigens in both microsatellite stable and unstable tumors, over two-thirds of which were derived from noncoding regions. Many of these peptides were derived from source genes known to be involved in colorectal cancer progression, suggesting that antigens from these genes could have therapeutic potential in a wide range of tumors. These findings could benefit the development of T cell–based vaccines, in which T cells are primed against these antigens to target and eradicate tumors. Such a vaccine could be used in tandem with existing immune checkpoint inhibition therapies, to bridge the gap in treatment efficacy across subtypes of colorectal cancer with varying prognoses. Data are available *via* ProteomeXchange with identifier PXD028309.

## Highlights

• Transcriptomic profiles highlight differences in colorectal cancer (CRC) subtypes.
• Normal adjacent tissues approximate “normal” expression of tumor-specific antigens.
• Over 30,000 MHC I–associated peptides identified from CRC cell lines and primary tumors.
• Proteogenomic analyses uncover 19 tumor-specific antigens.

## Graphical Abstract

**Figure:**
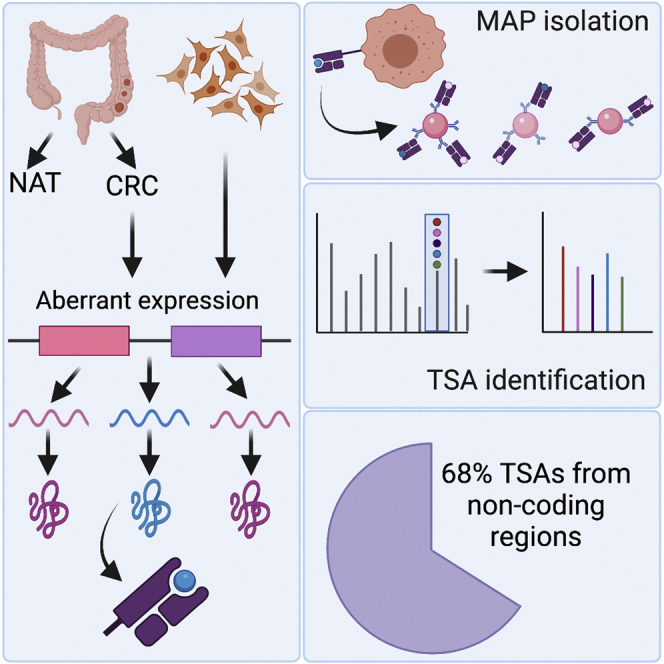

## In Brief

A novel proteogenomic approach was used to generate personalized cancer-specific databases and to identify more than 30,000 MHC I–associated peptides in colorectal cancer cell lines and primary tumors. Transcriptomic analyses were employed to distinguish microsatellite subtypes of colorectal cancer samples. This led to the first identification of aberrantly expressed tumor-specific antigens in colorectal cancer from both microsatellite stable and unstable tumors, all of which were demonstrably shown to be more abundant in tumors compared with normal adjacent tissues.

Colorectal cancer (CRC) is the third most commonly diagnosed cancer and the second leading cause of cancer death worldwide, with over 1.8 million cases and 881,000 deaths estimated in 2018 alone (1). The incidence of CRC is expected to increase as global socioeconomic changes occur, with a predicted 2.2 million cases and 1.1 million deaths occurring annually by 2030 (1, 2). This significant disease burden highlights the necessity of developing new and effective treatments.

The positive correlation between the abundance of tumor-infiltrating lymphocytes and increased overall survival in both colon and rectal cancer suggests that T cells can recognize biologically relevant tumor antigens in these tumors (3, 4). The potential immunogenicity of these antigens made immune checkpoint inhibition (ICI) a promising treatment for patients with cancer; however, early clinical trials evaluating their efficacy in CRC have yielded mixed results. Colorectal tumors characterized by deficiencies in mismatch repair proteins resulting in the accumulation of repetitive DNA sequences (microsatellites), known as microsatellite instability (MSI), have shown relative success in phase II clinical trials with anti-PD1 treatment (5). In contrast, such treatments have had very little efficacy in clinical trials against microsatellite stable (MSS) tumors that do not possess a high mutational burden, which make up approximately 80% of CRC cases (5, 6).

Given the significance of the immune response in CRC and the limited success of ICI alone, a promising research avenue in recent years has been neoantigen-based vaccines or T cell receptor–based therapy, which could be administered with ICI and would ideally bridge the gap in treatment efficacy across MSI and MSS tumors. In line with this, tumor-associated antigens (TAAs), which are overexpressed in cancer cells compared with normal cells, have been previously identified in CRC (7, 8). Although several TAAs have been tested in vaccine and phase I trials against CRC, most were met with “limited success,” likely due to the negative selection of TAA-responsive T cells in the thymus (9). In a study by Parkhurst *et al.*, the treatment of metastatic CRC with genetically engineered anti-carcinoembryonic antigen (CEA) T cells resulted in tumor regression in one individual but “serious inflammatory colitis” in all patients, demonstrating that an adverse autoimmune response is another possible consequence of targeting TAAs (10).

The mixed responses to TAA-based therapy suggest that targeting tumor-specific antigens (TSAs) would be more effective. These antigens may be generated through genetic, epigenetic, and posttranslational variations, including but not limited to single nucleotide variants, aberrantly expressed transcripts, or novel splicing events, and are exclusively presented by tumor cells (11). The high prevalence of single nucleotide variants, splice variants, and insertion/deletion (INDEL) mutations in CRC suggests that there is a higher probability of unique antigen presentation by the major histocompatibility complex (MHC) molecules of tumors compared with other cancers with lower mutational loads. These antigens, or MHC I–associated peptides (MAPs), would make it possible to invoke a tumor-specific immune response (12). TSAs have recently been identified in CRC and have demonstrated some success in phase I and II vaccine trials. A 2015 vaccine trial using frameshift antigens originating from MSI-high tumors demonstrated significant and specific immune responses among all patients (13). However, as this study used antigens derived from frameshift mutations associated with MSI, these findings do not apply to the majority of patients with CRC. Other studies identifying TSAs in CRC to date have focused exclusively on mutated TSAs (mTSAs) derived from coding regions of the genome (13, 14). An investigation of MSS CRC organoids revealed that only 0.5% of nonsilent mutations generated mTSAs; this was a significantly lower proportion than what was anticipated by human leukocyte antigen (HLA)-binding prediction software (15). It was recently demonstrated that the majority of actionable TSAs arise from noncoding regions of the genome and from aberrantly expressed transcripts, rather than somatic mutations (16, 17, 18). Although mTSAs are tumor specific unless derived from common driver mutations, these aberrantly expressed TSAs (aeTSAs) are particularly noteworthy because they may be shared by multiple tumors. In addition, previous studies did not employ mass spectrometry (MS) techniques to quantify the expression of those TSAs on tumor cells, which is information that could influence the therapeutic potential of targeting a given TSA (13, 14).

In the present study, we use an MS-based approach that leverages personalized databases to directly identify TSAs presented by CRC-derived cell lines and tumor biopsies and allows the identification of TSAs from noncoding regions. By using this approach, we identify 19 TSAs across our samples, as well as a variety of TAAs. Furthermore, we identify TSAs in both MSS and MSI tumors, suggesting that MSS tumors present immunologically relevant antigens that could be exploited to bridge the gap in treatment efficacy of ICI in various subtypes of CRC.

## Experimental Procedures

### Samples

#### Cell Lines

Four CRC cell lines (COLO 205 [ATCC CCL-222], HCT 116 [ATCC CCL-247], RKO [ATCC CRL-2577], SW620 [SW-620] [ATCC CCL-227]) and one normal fetal small intestine cell line (HIEC6 [ATCC CRL3266]) were obtained from the American Type Culture Collection (ATCC). A description of CRC-derived cell lines is presented in Table 1. COLO205, HCT116, and SW620 were grown in RPMI-1640 (Gibco) supplemented with 10% fetal bovine serum (FBS), RKO was grown in Eagle’s minimum essential medium (ATCC) supplemented with 10% FBS, and HIEC-6 was grown in OptiMEM 1 reduced serum medium (Gibco) supplemented with 20 mM Hepes (Gibco), 10 mM GlutaMAX (Gibco), 10 ng/ml epidermal growth factor (Gibco), and FBS to a final concentration of 4%. All cells were maintained at 37 °C with 5% CO_2_.

**Table 1:** Description of CRC-derived cell lines

Cell line	Tissue	Morphology	Disease	Biomarkers	MHC I molecule/cell	HLA genotyping	Mutations of interest
Colo205	Colon; derived from metastatic site: ascites	Epithelial	Dukes' type D, colorectal adenocarcinoma	MSS, CIMP	1.44 × 10^5^ ± 0.00282 × 10^5^	HLA-A∗01:01 HLA-A∗02:01	BRAF (V600E), SMAD4, TP53
						HLA-B∗07:02 HLA-B∗08:01	
						HLA-C∗07:01 HLA-C∗07:02	
HCT116	Colon	Epithelial	Colorectal carcinoma	MSI, CIMP	5.07 × 10^5^ ± 0.30 × 10^5^	HLA-A∗01:01 HLA-A∗02:01	RAS (G13D), PI3CA, CDKN2A, CTNNB1 (B-catenin)
						HLA-B∗18:01 HLA-B∗45:01	
						HLA-C∗05:01 HLA-C∗07:01	
RKO	Colon	Epithelial	Carcinoma	MSI, CIMP	2.82 × 10^5^ ± 0.11 × 10^5^	HLA-A∗03:01	BRAF (V600E), PI3CA
						HLA-B∗18:01	
						HLA-C∗07:01	
SW620	Colon; derived from metastatic site: lymph node	Epithelial	Dukes' type C, colorectal adenocarcinoma	MSS, CIN	1.69 × 10^5^ ± 0.0017 × 10^5^	HLA-A∗02:01 HLA-A∗24:02	APC, RAS (G12V), SMAD4, TP53
						HLA-B∗07:02 HLA-B∗15:18	
						HLA-C∗07:02 HLA-C∗07:04	

For collection, cells were rinsed with warm phosphate-buffered saline (PBS) before being trypsinized with TrypLE Express Enzyme (1×) (Gibco) for 5 to 15 min at 37 °C with 5% CO_2_. Harvested material was then spun at 1000 rpm for 5 min, rinsed once with warm PBS, then resuspended in ice-cold PBS. After cell count, replicates of 2 × 10^8^ CRC cells were pelleted and frozen at −80 °C until further use. MHC class I surface density of the CRC cell lines was determined by Qifikit (Agilent) using the W6/32 anti-HLA class I antibody (BioXCell), according to the manufacturer’s instructions.

#### Primary Tissues

Six pairs of primary human samples consisting of matched colon adenocarcinoma tumor and normal adjacent tissue (NAT) were purchased from Tissue Solutions. Tissue samples were taken from patients receiving surgery as the first line of treatment and were flash frozen in liquid nitrogen. More information about primary tissue samples can be found in Table 2.

**Table 2:** Description of primary tumor and matched NAT

Sample ID	Sex	Age	Ethnic background	Matrix	Diagnosis	Histological diagnosis	Stage	Tumor content %	Mutations of interest	HLA
S1_N				colon	NAT					HLA-A∗24:02
S1_T	F	73	Caucasian	Cecum	Cancer	adenocarcinoma	IIC	100	KRAS G12D	HLA-B∗07:02 HLA-B∗35:01
										HLA-C∗04:01 HLA-C∗07:02
S2_N				Colon	NAT					HLA-A∗02:01 HLA-A∗03:02
S2_T	M	60	Caucasian	Sigmoid	Cancer	Adenocarcinoma	IIA	95		HLA-B∗27:05 HLA-B∗58:01
										HLA-C∗02:02 HLA-C∗07:01
S3_N				Colon	NAT					HLA-A∗01:01 HLA-A∗32:01
S3_T	F	63	Caucasian	Sigmoid	Cancer	Adenocarcinoma	IIA	100	KRAS Q61H	HLA-B∗38:01 HLA-B∗50:01
										HLA-C∗06:02 HLA-C∗12:03
S4_N				Colon	NAT					HLA-A∗01:01 HLA-A∗11:01
S4_T	F	85	Caucasian	Sigmoid	Cancer	Adenocarcinoma	IIA	100	KRAS G12D	HLA-B∗15:01 HLA-B∗57:01
										HLA-C∗03:03 HLA-C∗06:02
S5_N				Colon	NAT					HLA-A∗03:01 HLA-A∗30:01
S5_T	F	43	Caucasian	Ascending colon	Cancer	Adenocarcinoma	IIA	95		HLA-B∗13:02 HLA-B∗52:01
										HLA-C∗06:02 HLA-C∗12:02
S6_N				Colon	NAT					HLA-A∗03:01 HLA-A∗23:01
S6_T	F	48	Caucasian	Sigmoid	Cancer	Adenocarcinoma	IIA	95		HLA-B∗07:02 HLA-B∗18:01
										HLA-C∗07:01 HLA-C∗07:02

### RNA Extraction and Sequencing

#### RNA Extraction

For RNA extraction of cell lines, 1 to 2 million cells were collected and washed once with ice-cold PBS. The cells were then resuspended in Trizol (Invitrogen). Total RNA was isolated using the RNeasy Mini kit (Qiagen) or the AllPrep DNA/RNA/miRNA Universal kit (Qiagen) as recommended by the manufacturer, for cell lines and tissues, respectively.

#### RNA Sequencing

Five hundred nanograms of total RNA was used for library preparation. RNA quality control was assessed with the Bioanalyzer RNA 6000 Nano assay on the 2100 Bioanalyzer system (Agilent Technologies), and all samples had an RNA integrity number (RIN) above 6.8 for NAT and above 8 for cancer samples. Libraries were prepared with the KAPA mRNAseq Hyperprep kit (Roche). Ligation was made with Illumina dual-index UMI (IDT). After being validated on a BioAnalyzer DNA1000 chip and quantified by QuBit and qPCR, libraries were pooled to equimolar concentration and sequenced with the Illumina Nextseq500 using the Nextseq High Output 150 (2 × 75 bp) cycles kit. A mean of 129 and 95 million paired-end PF reads were generated for the cell lines and tissue samples, respectively. Library preparation and sequencing were performed at the Genomic Platform of the Institute for Research in Immunology and Cancer (IRIC).

#### Bioinformatic Analyses

Sequences were trimmed using Trimmomatic version 0.35 (19) and aligned to the reference human genome version GRCh38 (gene annotation from Gencode version 33, based on Ensembl 99) using STAR version 2.7.1a (20). Gene expressions were obtained both as read count directly from STAR as well as computed using RSEM (21) to obtain normalized gene and transcript-level expression, in transcript-per-million (TPM) values, for these stranded RNA libraries.

### Transcriptomics

#### HLA Genotyping

HLA genotyping of cell lines and tissues was performed using OptiType, an online HLA genotyping tool that uses RNA-Seq data to predict a sample’s HLA alleles (https://github.com/FRED-2/OptiType) (22). HLA alleles of cell lines were confirmed with what is documented in the literature, and if these differed from Optitype predictions, we preferentially selected those in the literature.

#### Microsatellite Instability Detection

MSI status of the primary tumor samples was evaluated using the MSIsensor-pro1.0a program using paired tumor and NAT (https://github.com/xjtu-omics/msisensor-pro) (23).

#### Differential Expression Analysis

DESeq2 version 1.22.2 (24) was used to normalize gene read counts. Principal component analyses were generated using normalized log read counts for the first two most significant components. The principal component analysis was generated in an unsupervised manner. The 500 genes were those presenting the biggest standard deviation based on their expression levels across all samples. DESeq2 was only used to normalize the read counts, not to perform a differential expression analysis. For differential expression analysis of the cell lines, fold changes were computed between the mean expression of the four CRC cell lines compared with the normal cell line (HIEC-6). Significant differentially expressed genes (DEGs), those with padj <0.05 and ∣log2 fold change∣ >1, were considered for gene ontology (GO) terms using the Metascape tool (25). For paired differential expression analysis of the tissues, TPM normalized values were used to compare tumor/NAT pairs. As only a single replicate of the tissues was sequenced, rather than filtering by adjusted *p*-value, we selected only genes that were significantly differentially expressed in all six subjects for GO term analysis with ∣log2 fold change∣ >1. When examining DEGs between MSS and MSI tissues, the same fold change thresholds were applied. For GO term analysis of MSI DEGs, genes were selected that were exclusively differentially expressed in both MSI tissues (*i.e.*, not considered DEGs in any MSS tissues). For GO term analysis of MSS DEGs, genes were considered if they were differentially expressed in three or more MSS tissues.

#### Transcriptome Analysis of Tissue Samples

The proportion of various biotypes in the transcriptome of tissue samples was determined as described (26). Briefly, following quantification and alignment of Ensembl annotated transcripts by Kallisto (21), transcripts and repetitive elements were annotated using a Kallisto index containing Ensembl annotated transcripts supplemented with genetic repeat identifications from the UCSC Table Browser GRCh38 repeat masker database (27). Transcript expression was quantified in TPM.

#### Mutation Profiles/“Genetic Variant Annotation”

Genetic variant calling was performed for both cell line and primary biopsies using SNPEff (https://pcingola.github.io/SnpEff/#snpeff) (28).

### Database Generation

Global cancer databases were constructed as described (16). In brief, RNA-Seq reads were trimmed using Trimmomatic version 0.35 (19) and aligned to the reference human genome version GRCh38 (gene annotation from Gencode version 33, based on Ensembl 99) using STAR version 2.7.1a (20). Kallisto (https://pachterlab.github.io/kallisto) was used to quantify transcript expression in TPM (21). Sample-specific exomes were constructed by integrating single nucleotide variants (quality >20) identified with Freebayes (https://github.com/ekg/freebayes) into PyGeno (29). Annotated open reading frames with TPM > 0 were then translated *in silico* and added to the canonical proteome in fasta format. We selected medullary thymic epithelial cells (mTECs) and thymic epithelial cells (TECs) as a positive control because they (1) express a large collection of self-peptides and (2) establish central tolerance in the thymus (negative selection of T cells). mTECs (n = 6) and TECs (n = 2) were thus used to generate the cancer-specific proteome for cell lines (GEO accessions GSE127825, GSE127826). The respective NAT for each primary tumor sample was used in place of mTECs for this portion of database construction, as it approximates “normal” expression for that subject. RNA-Seq reads were cut into 33-nucleotide sequences known as k-mers and only k-mers present <2 in mTECs or matched NAT for cell lines and tissues, respectively, were kept. Overlapping k-mers were assembled into contigs, which were then three-frame translated *in silico*. Of note, short peptide sequences generated through the k-mer approach were then concatenated into longer sequences of approximately 10,000 amino acids. To reduce the number of small separate sequences in the cancer-specific proteome, these peptides were concatenated using the “JJ” sequence as a separator, which is recognized internally by the PeaksX+ software to split sequences upon occurrence of this sequence. Then, the canonical proteome and the cancer-specific proteome were concatenated to create the global cancer databases. Cell line databases consisted of 3.38 × 10^6^ sequences on average.

### Isolation of MAPs

CRC cell line pellet samples (2 × 10^8^ cells per replicate, four replicates per cell line) were resuspended with PBS up to 2 ml and then solubilized by adding 2 ml of ice-cold 2× lysis buffer (1% w/v CHAPS). Tumor and NAT samples (average 568 mg) were cut into small pieces (cubes, ∼3 mm in size), and 5 ml of ice-cold PBS containing protein inhibitor cocktail (Sigma, cat#P8340-5 ml) was added. Tissues were first homogenized twice for 20 s using an Ultra Turrax T25 homogenizer (IKA-Labortechnik) set at a speed of 20,000 rpm and then 20 s using an Ultra Turrax T8 homogenizer (IKA-Labortechnik) set at speed 25,000 rpm. Then, 550 μl of ice-cold 10× lysis buffer (5% w/v CHAPS) was added to each sample. After 60-min incubation with tumbling at 4 °C, tissue samples and CRC cell line samples were spun at 10,000*g* for 30 min at 4 °C. Supernatants were transferred into tubes containing 1 mg of W6/32 antibody covalently cross-linked protein A magnetic beads, and MAPs were immunoprecipitated as described (30). MAP extracts were then dried using a Speed-Vac and kept frozen before MS analyses.

### TMT Labeling

MAP extracts were resuspended in 200 mM HEPES buffer pH 8.1. Tandem mass tag (TMT) reagent (Thermo Fisher Scientific), 50 μg, in anhydrous acetonitrile was added to samples as follows: CRC cell line replicates were labeled with TMT6plex (lot #UG287166) channels TMT6-126 to 129; tissue samples were labeled with TMT10plex (lot # UH285228) -126 (NAT) and -127N (tumor). Samples were gently vortexed and reacted at room temperature for 1.5 h. Samples were then quenched with 50% hydroxylamine for 30 min at room temperature, then were diluted with 4% formic acid (FA) in H_2_O. CRC cell line replicates and individual NAT-tumor pairs were combined. Samples were then desalted on homemade C18 membrane (Empore) columns and stored at −20 °C until injection. Labeling efficiency was calculated using PeaksX+ search results (see “MAP Identification” section below), by taking the proportion of TMT peptide spectrum matches (PSMs) over the total number of PSMs.

### Mass Spectrometry Analyses

Dried peptide extracts were resuspended in 4% FA and loaded on a homemade C18 analytical column (20 cm x 150 μm i.d. packed with C18 Jupiter Phenomenex) with a 106-min gradient from 0% to 30% ACN (0.2% FA) and a 600-nl/min flow rate on an EASY-nLC II system. Samples were analyzed with an Orbitrap Exploris 480 spectrometer (Thermo Fisher Scientific) in positive ion mode with Nanoflex source at 2.8 kV. Each full MS spectrum, acquired with a 240,000 resolution was followed by 20 MS/MS spectra, where the most abundant multiply charged ions were selected for MS/MS sequencing with a resolution of 30,000, an automatic gain control target of 100%, an injection time of 700 ms, and collisional energy of 40%. The LC-MS instrument was controlled using Xcalibur version 4.4 (Thermo Fisher Scientific, Inc).

### MAP Identification

Database searches were conducted using the PeaksX+ software, version 10.6 (Bioinformatics Solutions Inc) (31). Error tolerances for precursor mass and fragment ions were set to 10.0 ppm and 0.01 Da, respectively. A nonspecific digest mode was used. TMT10plex was set as a fixed posttranslational modification, and variable modifications included phosphorylation (STY), Oxidation (M), Deamidation (NQ), and TMT10plex STY. Peaks searches were then loaded into MAPDP (32), which was used to apply the following filters: selecting peptides of 8 to 11 amino acids in length, with rank eluted ligand threshold ≤2% based on NetMHCpan-4.1 b predictions, using a 5% false discovery rate (FDR). FDR was calculated using the decoy hits imported from Peaks, which employ the decoy-fusion strategy (33).

#### MAP Source Gene Analysis

GO term analysis was performed for CRC-derived cell lines and primary tissues with the Metascape tool (25). A list of source genes was generated for each sample by taking all of the source genes associated with the MHC I immunopeptidome of that set of samples and removing duplicates (*i.e.*, although a source gene may generate more than one unique peptide, it would only be counted once in the source gene analyses). For tissues, only source genes shared by four or more tissues were included in this analysis.

### Quantification of MAP Coding Sequences in RNA-Seq Data

MAP-coding sequences (MCSs) were quantified in RNA-Seq data as described (18). Briefly, MCSs were reverse translated into all possible nucleotide sequences with an in-house python script (deposited on Zenodo at DOI: 3739257). The nucleotide sequences were then mapped onto the genome with GSNAP (34) to determine all possible genomic locations able to code for a given MAP. MCSs were also mapped onto the transcriptome to account for MAPs overlapping splice sites, and portions of the transcriptome corresponding to these MAPs were then also mapped onto the reference genome with GSNAP. For MAPs of interest, we performed genomic alignment of all reads containing the MCS. GSNAP output was filtered to keep only perfect matches between the sequence and reference, resulting in a file containing all possible genomic regions able to code for a given MAP. We summed the number of reads containing the MCSs at their respective genomic locations in each desired RNA-Seq sample (such as CRC and NAT, Genotype Tissue Expression project [GTEx], or the Cancer Genome Atlas [TCGA] samples), aligned on the reference genome with STAR. Lastly, all read counts for a given MAP were summed and normalized on the total number of reads sequenced in each sample of interest to obtain a reads-per-hundred-million (RPHM) count.

### Determination of MAP Source Transcripts

To investigate what proportion of tissue sample MAPs were derived from certain transcript biotypes, the most abundant putative source transcript based on kmer-per-hundred-million (KPHM) quantification was determined. For peptides from the cancer-specific (kmer) database, the MCSs were reverse translated into all possible nucleotide sequences and all possible genomic regions able to code for a given MAP were identified (see “Quantification of MAP Coding Sequences in RNA-Seq Data” above). Finally, Kallisto was used to determine the most expressed transcript at that location, which was then assigned as the most probable transcript for the given peptide. Peptides that had more than one putative source transcript were excluded from the analysis.

### Identification of TSA Candidates

TSA candidates were identified through a stringent TSA identification pipeline. First, MAPs underwent peptide classification in which the peptide sequence accessions were retrieved from the protein database and used to extract the nucleotide sequences of each peptide. RNA-Seq data from each cancer and normal samples were transformed into 24-nucleotide-long k-mer databases with Jellyfish 2.2.3 (using the –C option) and used to query each TSA candidate coding sequence’s 24-nucleotide-long k-mer set. The number of reads fully overlapping a given peptide-coding sequence was estimated using the k-mer set’s minimum occurrence (kmin), as in general, one k-mer always originates from a single RNA-Seq read. We then transformed this *kmin* value into several k-mers detected per 10^8^ reads sequenced (kphm) using the following formula: kphm = (*kmin* × 10^8^)/rtot, with rtot representing the total number of reads sequenced in a given RNA-Seq experiment. Peptides were kept only if their RNA coding sequences were expressed at least 10-fold higher in cancer than in normal (pooled mTEC samples for cell lines, matched NAT for tissues) and expressed <2 KPHM in normal. Subsequent filtering removed any peptides with indistinguishable isoleucine/leucine variants; a peptide with an IL variant was kept only if the most expressed variant met the above-mentioned criteria. The MCSs of the remaining peptides were quantified in RNA-Seq data as described above and were kept only if their expression was <8.55 RPHM in mTECs and other normal tissues (GTEx). Genomic localization for each peptide was assigned by mapping reads containing each MCS to the reference genome (GRCh38.99) using BLAT (https://genome.ucsc.edu/cgi-bin/hgBlat). Peptides were excluded if the genomic localization was unclear or if they mapped to a hypervariable region (HLA, Ig, or T cell receptor genes). Finally, the MS/MS spectra of the remaining candidates were manually validated. Peptides were classified as mTSAs if their amino acid sequence was different from the reference and if the mutation was not a known germline polymorphism. Peptides were classified as aeTSAs if they were overexpressed ≥10-fold in tumor compared with normal and ≤0.2 KPHM in mTECs (and NAT in the case of tissues) and as TAAs if they were overexpressed ≥10-fold in cancer but the expression in mTECs and/or NAT was >0.2 KPHM. Ultimately, the transcript of origin of each TSA/TAA was attributed by selecting the most highly expressed peptide-overlapping transcript from the kallisto quantification file (see “Database Generation” section).

#### Intertumoral Sharing

To examine the intertumoral distribution of TSA and TAA sequences in other CRC tumors, the log(RPHM+1) expression of the peptide coding sequences in 151 colon adenocarcinoma (COAD) samples from TCGA was determined (see “Quantification of MAP Coding Sequences in RNA-Seq Data”).

#### Immunogenicity Prediction

The predicted immunogenicity of MAPs of interest was determined with the R package Repitope v3.0.1 (https://github.com/masato-ogishi/Repitope) (35).

### TSA Validation and Relative Quantification With Synthetic Peptides

#### Validation of TSA Peptide Candidates

Synthetic peptides of TSA and select TAA sequences were obtained from Genscript. Synthetic peptides were solubilized in dimethyl sulfoxide to a concentration of 1 nmol/μl, and all synthetic peptides were combined in a stock solution at a concentration of 10 pmol/μl. The stock solution was desalted in aliquots of 150 pmol on homemade C18 membrane (Empore) columns and dried using a Speed-Vac. Dried peptide extracts were labeled with a TMT10plex channel as described (see “TMT Labeling” section), desalted, and dried down in Speed-Vac. Labeled synthetic peptides were resuspended in 4% FA, and 1 pmol of each synthetic peptide was loaded on a homemade C18 analytical column (20 cm x 150 μm i.d. packed with C18 Jupiter Phenomenex) with a 76-min gradient from 0% to 30% ACN (0.2% FA) and a 600-nl/min flow rate on an EASY-nLC II system. Samples were analyzed with an Orbitrap Exploris 480 spectrometer (Thermo Fisher Scientific) in positive ion mode with Nanoflex source at 2.8 kV. Each full MS spectrum was acquired with a 120,000 resolution, and an inclusion list was used to select ions for fragmentation with 40% collision energy and an isolation window of 1 m/z. MS/MS were acquired with a resolution of 30,000. MS/MS correlations were computed as described (17). Briefly, expected peptide fragments were computed with pyteomics v4.0.1 (https://bitbucket.org/levitsky/pyteomics) and reproducibly detected peptide fragments were identified. Root scaled intensities of these fragments were correlated between endogenous and synthetic peptide scan pairs, and SciPy v1.2.1 (https://www.scipy.org/) was used to compute Pearson correlation coefficient, *p*-value, and confidence intervals. Mirror plots of the scan pair with the lowest *p*-value were generated for each peptide using spectrum_utils v0.2.1(https://github.com/bittremieux/spectrum_utils).

#### Relative Quantification

To relatively quantify MAPs of interest in primary tissue samples, synthetic peptides at concentrations of 10, 100, or 1000 fmol labeled with TMT 10plex-129N, 130N, and 131N, respectively, were spiked into remaining purified MAPs from NAT and CRC tissue samples labeled with TMT10plex-126 and 127N, respectively. Note that the channel TMT 10plex-127C was left empty to assess contamination. Samples were analyzed with an Orbitrap Fusion Tribrid spectrometer (Thermo Fisher Scientific) in positive ion mode with Nanoflex source at 2.4 kV. For synchronous precursor selection MS3 (SPS-MS3), full MS scans were acquired with a range of 300 to 1000 *m/z*, Orbitrap resolution of 120,000, automatic gain control (AGC) of 5.0e5, and a maximum injection time of 50 ms, using an inclusion list for the peptides of interest. We used a 3s top speed approach for MS2 in the ion trap, with an isolation window of 0.4 *m/z*, collision induced dissociation of 35%, a “normal” ion trap scan rate mode, 2.0e4 AGC target, and 50 ms maximum injection time. This was followed by the selection of eight synchronous precursor ions for MS3 acquisition, which was done with a scan range of 110 to 500 *m/z*, Orbitrap resolution of 50,000, AGC of 1.0e5, maximum injection time of 300 ms, an isolation window of 2.0 *m/z*, and 65% HCD collision energy. LC-MS instrument was controlled using Xcalibur version 4.4 (Thermo Fisher Scientific, Inc). Error tolerances for precursor mass and fragment ions were set to 10.0 ppm and 0.5 Da, respectively. A nonspecific digest mode was used. TMT10plex was set as a fixed posttranslational modification, and variable modifications included phosphorylation (STY), Oxidation (M), Deamidation (NQ), and TMT10plex STY. For quantification, PSMs were filtered to exclude those with contamination in the TMT10plex-127C channel and to select those within the 70th intensity percentile. MS2 precursor profiles and intensity profiles of all relevant channels were manually inspected to select peptides for quantification. Intensity ratios for each peptide were calculated using the average TMT10plex-127N and TMT10plex-126 intensities of good-quality PSMs.

### Data Analysis and Visualization

**Fig. 1:**
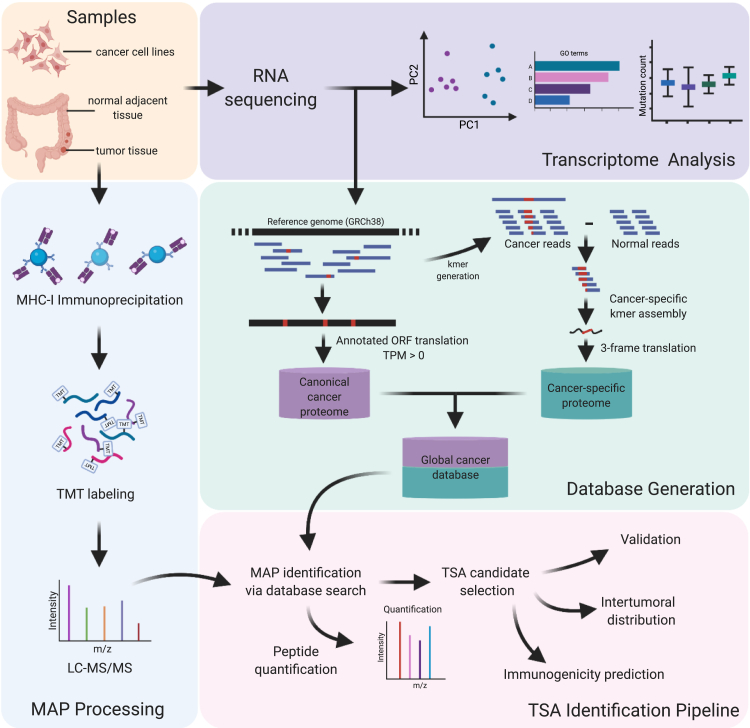
**Proteogenomic workflow for the discovery of tumor-specific antigens (TSAs) in both colorectal cancer–derived cell lines and primary tumor samples**. Samples generated from colorectal cancer– and normal intestine-derived cell lines and matching primary tumor/normal adjacent tissue biopsies obtained from six individuals were all processed for both RNA sequencing and major histocompatibility complex class I (MHC-I) immunoprecipitation. RNA sequencing data were used for both the transcriptomic characterization of the samples and the generation of customized global cancer proteome databases. For each sample, the MHC-I associated peptides (MAPs) isolated *via* immunoprecipitation were identified *via* LC-MS/MS using the respective database. After validating both the identification and the tumor specificity of our TSA candidates, their therapeutic potentials were evaluated through the prediction of both their immunogenicity and intertumoral distribution. Created with BioRender.com.

Figure 1 and the visual abstract were generated with BioRender.com. The majority of other figures were created with Python v3.7.6, R v3.6.3, or Origin (Pro)2019b. R packages include:

Repitope v3.0.1 (https://github.com/masato-ogishi/Repitope) (35),

UpsetR v1.4.0 (https://github.com/hms-dbmi/UpSetR) (36),

GSVA v1.38.2 (https://github.com/rcastelo/GSVA) (37),

ESTIMATE v1.0.13 (https://bioinformatics.mdanderson.org/estimate/) (38).

### Experimental Design and Statistical Rationale

To effectively elucidate the MHC I immunopeptidome of colorectal cancer, four CRC cell lines and six samples from human subjects consisting of both matched tumor and NAT were selected. NAT was used as an approximation of healthy tissue, as it is the most effective control for each respective tumor. Since no matched samples were available for cell lines, a pool of eight TEC samples was used in the creation of global cancer databases, to obtain a wide berth of approximate normal RNA expression. All instances of *p*-values are determined using two-sample *t* test, except in the determination of significance for immunogenicity scores, in which case the Mann–Whitney test was used as the data did not have a normal distribution, as determined by the Shapiro test. For *t* tests, we performed f-tests to determine whether the dataset had significant variation; if yes, then we used the *t* test assuming variation, and otherwise the *t* test assuming no variation was used. For CRC-derived cell lines, four technical replicates of 2 × 10^8^ cells were prepared, which were TMT labeled and multiplexed prior to injection. Owing to limited tissue material, half of the purified MAPs from primary samples were injected to obtain global immunopeptidomic data and the remaining sample was used for targeted analysis with synthetic peptides to confirm the sequences and abundance of putative TSAs and select TAAs. To select high-quality PSMs for quantification, those of low intensity or with contamination in an empty TMT channel were excluded. Furthermore, only peptides with favorable MS2 precursor and intensity profiles were quantified.

## Results

### Immunopeptidomic Analyses Using a Proteogenomic Approach

To determine the composition of the immunopeptidome of CRC, we analyzed a collection of samples comprising four CRC-derived cell lines and six sets of primary adenocarcinoma samples, which consist of matched tumor and NAT (Tables 1 and 2). Paired-end RNA-Seq allowed the creation of a global cancer database, consisting of a canonical cancer proteome as well as a cancer-specific proteome for each sample, by generating cancer-specific kmers, which, once combined into contigs, are translated into three reading frames to encompass noncanonical sequences from any genomic origin (Fig. 1, green box). mTECs present peripheral antigens in the thymus and mediate the negative selection of autoreactive T cells (39). Thus, in the case of CRC-derived cell lines, cancer-specific kmers were obtained following the subtraction of mTEC-derived kmers, which approximated the expression of these sequences in healthy tissues. For the primary tissue samples, the cancer-specific kmers were generated following subtraction of the sequences from matched NATs. This approach enabled the determination of sequences expressed in tumor and not observed in healthy colon tissue of the same individual. In addition to database construction, RNA-Seq data were also used for transcriptomic analysis, including GO term analysis, investigation of immune infiltration, mutation profiling, and determination of transcript abundance (Fig. 1, purple box).

We used immunoprecipitation to isolate MHC I–peptide complexes, and we labeled the eluted MAPs with TMT isobaric labeling reagent, as TMT labeling was recently shown to enhance the detection of MAPs by increasing their charge state and hydrophobicity (Fig. 1, blue box) (40). We then sequenced and analyzed MAPs by liquid chromatography tandem mass spectrometry (LC-MS/MS) and identified using the personalized cancer databases generated through RNA-Seq. Identified MAPs then underwent a rigorous series of classifications and validations to identify putative TSAs and TAAs. Tumor antigens identified in CRC tissues were then validated and quantified with synthetic peptides to determine to what extent they were overexpressed on tumor compared with matched NAT, and we also investigated their predicted immunogenicity and intertumoral distribution to evaluate their clinical potential (Fig. 1, pink box). The TSA and TAA selection process was composed of a stringent set of filters based on expression in cancer and normal tissues (supplemental Fig. S1).

We used four CRC-derived cell lines with different HLA alleles and characteristics as summarized in Table 1. HCT116 and RKO are derived from primary tumors and are characterized by MSI, whereas Colo205 and SW620 are derived from metastases of ascites and lymph node, respectively, and are both MSS. Among the 4 cell lines are mutations in several key genes, such as BRAF, RAS, SMAD4, TP53, and PI3CA. These cell lines have a varying MHC I surface expression ranging from 1.44 × 10^5^ to 5.07 × 10^5^ MHC I molecules/cell, as determined by Qifikit, and a diversity of HLA alleles, which were identified using OptiType, an HLA genotyping tool that uses RNA-Seq data to predict a sample’s HLA alleles, in combination with the HLA alleles for these cell lines documented in the literature (Table 1) (22).

All of the primary tumor samples are derived from stage II adenocarcinomas, which vary only slightly in tumor grade and TNM (tumor-node-metastases) classification (Table 2). The CRC tissue samples had a tumor content of 95% to 100% and an average mass of 0.6625 g. The tumors all originated from the sigmoid colon, with the exception of S1 (cecum) and S5 (ascending colon). NAT were collected at least 6 cm away from the tumor margins. Similar to the cell lines, the tissue samples also possess a variety of HLA alleles. A visualization of the number of HLA alleles unique to or shared by cell line and tissue samples is available in supplemental Fig. S2. There is an average of 1.3 and 3.2 unique alleles per cell line and tissue, respectively.

## Transcriptomic Analyses Reveal Heterogeneity Between MSI and MSS Samples

Because the outcome for patients with CRC within a given disease stage differs greatly based on the molecular characteristics of the tumor (41, 42), RNA sequencing data were used to characterize the molecular heterogenicity of the samples. After first examining the mutational status of key biomarkers (such as KRAS, NRAS, or BRAF), which are commonly used to guide therapeutic decisions and prognoses in the clinics (43, 44) (Table 1), the microsatellite statuses of cell lines and primary samples were, respectively, determined from the literature (45, 46) and using the MSIsensor package (47). Although MSI is found in a limited subset of CRC tumors (*i.e.* 15% of sporadic CRC and 90% of nonpolyposis CRC) (48), in this study, 50% of the tumorigenic cell lines and 33% of the primary biopsies present this phenotype (supplemental Table S1). Although several elements in the literature suggest that MSI and MSS tumors are immunologically different (5, 11, 49, 50), this study will provide the first comparison of MSI and MSS colorectal tumors at the immunopeptidomic level.

**Fig. 2:**
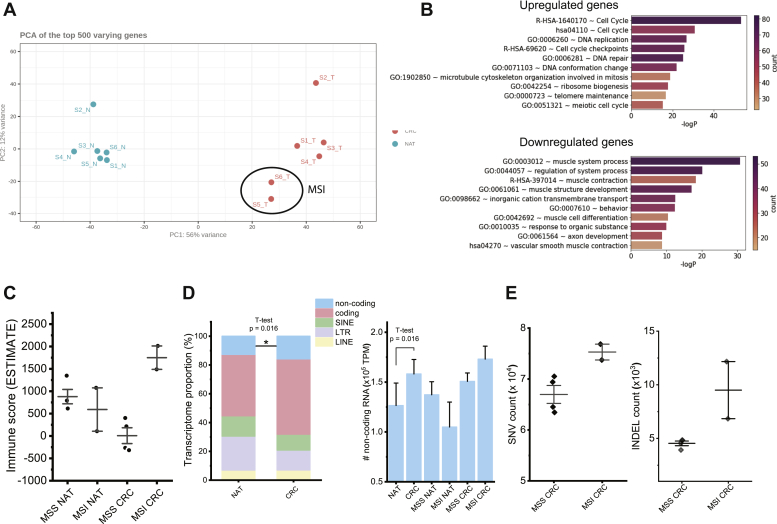
**Transcriptomic profile of primary tumor/normal adjacent tissue CRC biopsies**. *A*, principal component analysis (PCA) of the top 500 varying genes of each tumor/NAT sample following paired-end RNA-Seq and gene read count normalization with DESeq2. MSI tissues (as determined by MSISensor) are encircled. *B*, GO term analysis of genes up/downregulated in CRC tissues compared with their adjacent NAT. Genes submitted to GO term analysis were those with ∣log2FC∣ >1 and that were found to be differentially regulated in all samples, using TPM normalized values. *C*, bar graph showing the mean ESTIMATE immune score of MSS NAT, MSI NAT, MSS CRC, and MSI CRC, with standard deviation shown. *D*, stacked bar graph showing the mean proportion of the transcriptome attributable to five distinct transcript biotypes in NAT *versus* CRC samples, with the differences in the proportion of noncoding transcripts being statistically significant between NAT and CRC (noncoding: *p* = 0.016; coding: *p* = 0.078; SINE: *p* = 0.15; LTR: *p* = 0.056; LINE: *p* = 0.95). *E*, scatterplots displaying the SNV counts and INDEL counts of MSS and MSI CRC tissues determined by SNPEff genomic annotation, with mean and standard error bars. CRC, colorectal cancer; MSI, microsatellite instability; MSS, microsatellite stable; NAT, normal adjacent tissue; TPM; transcripts per million.

Principal component analysis of the top 500 varying genes between normal and tumor biopsy samples (Fig. 2*A*) or cell lines (supplemental Fig. S3*A*) confirms their distinct transcriptomic profile. Accordingly, pathway and process enrichment analysis of biopsy samples revealed a transcriptomic profile enriched in terms associated with their tumorigenic status (Fig. 2*B*). As expected for CRC, the most significantly up- and downregulated terms are, respectively, linked to cell proliferation (Fig. 2*B* upper panel) and muscle phenotype and contractility (Fig. 2*B* lower panel). Although the enrichment of terms related to proliferation and cell cycle is a general hallmark of cancer (51, 52), the downregulation of muscle-related pathways is inherent to CRC and results from the functional dichotomy between poorly differentiated tumor areas and highly contractile NAT. In contrast, intertumoral transcriptomic differences were mostly explained by the MSI/MSS status of the tumor samples of our datasets (Figs 2*A* and S3*A*). Although MSI samples tend to cluster tightly together, MSS tumors appear more dispersed and therefore transcriptionally more heterogeneous. Functionally, when analyzed separately, the MSS and MSI CRC samples are enriched in very different gene sets. When compared with their corresponding NAT, MSI tumors are characterized by a significant upregulation of various immune-related terms (supplemental Fig. S3*B*), whereas MSS tumors are more associated with an increased expression of genes related to both Wnt and PI3K-Akt signaling (supplemental Fig. S3*C*). Although the link of these two signaling pathways with CRC is well established in the literature (53), no reference could be found to support that their contribution in CRC may differ between MSS and MSI tumors.

Next, we estimated the degree of immune infiltration of each sample *via* two independent approaches using the immune infiltration score from the ESTIMATE package (38) (Fig. 2*C*), and with an enrichment score for known tumor-infiltrating leukocyte (TIL) markers (54) based on a single-sample Gene Set Enrichment Analysis (55) (supplemental Fig. S4*A*). Although all NAT samples presented similar levels of immune infiltration, MSI and MSS tumors were, respectively, characterized by increased and decreased immune infiltration scores (Figs. 2*C* and S4*A*). Consistent with what has been previously reported in the literature (49, 56, 57, 58, 59), such differences suggest that MSI tumors may be more immunogenic than their MSS homologs.

Because TSAs can arise from a wide range of cancer-specific events/dysregulations (11, 12) and the immunopeptidome contribution of each antigenic source varies significantly across malignancies (11), RNA-Seq data were also used to inform which TSA classes might be enriched in our samples. By examining the genomic origin of the transcripts, we observed that both the proportion and the absolute abundance of noncoding polyadenylated RNAs are significantly increased in tumors compared with NATs (Fig. 2*D*). Although we cannot exclude the possibility that the higher proportion of noncoding RNAs could at least in part reflect the different cellular composition of the tumor and NAT samples, it is well documented that noncoding RNAs are frequently deregulated in cancer (60), and specifically in colon cancer (61). In addition, although the average absolute abundance increase is limited to 25%, our data suggest that the tumor-specific gain of noncoding transcripts could be higher in MSI tumors than in MSS. Although this comparison remains limited due to the low number of MSI samples (n = 2), one could expect to identify a higher number of aeTSA deriving from noncoding transcripts in MSI samples compared with MSS. Both the single nucleotide variant (SNV) burden and the INDEL burden are notably increased in MSI samples compared with MSS (an average difference of 8326 and 4965 between MSI and MSS mean SNV and INDEL burdens, respectively), an observation that is also noted for cell lines (Figs 2*E* and S4*B*). Considering both cell line and tissue samples together resulted in a statistically significant difference in the number of INDEL mutations between MSS and MSI samples (*p* = 0.0024) (supplemental Fig. S4*C*). Because both MSI and INDEL accumulations result from defects in the DNA mismatch repair pathway (62), one can hypothesize that the number of INDEL-derived TSAs (most likely frameshift-derived antigens) identified in a sample will be proportional to its MSI level.

## Immunopeptidomic Analyses Highlight the Diversity of CRC Antigens

**Fig. 3:**
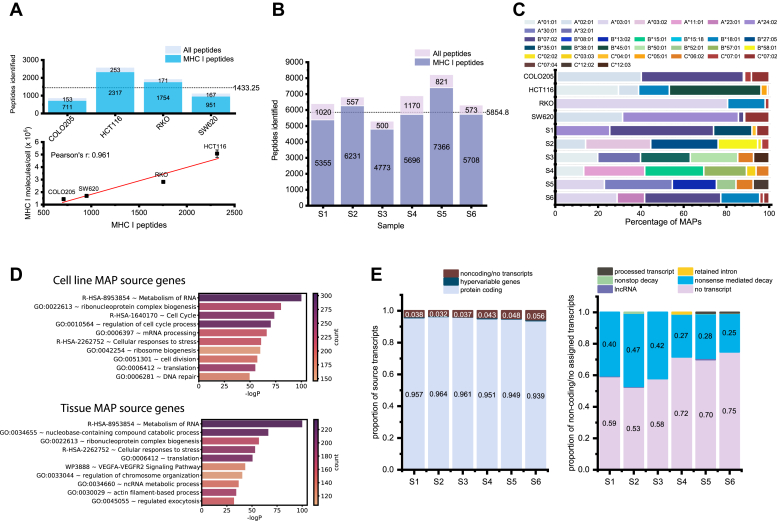
**Immunopeptidomics of CRC-derived cell lines and tissues.***A*, t*op panel*: Stacked bar chart displaying the number of unique peptides identified in CRC cell lines, and a horizontal line indicating the average number of MAPs per cell line. *Bottom panel*: Scatterplot indicating the correlation between the number of unique MAPs identified in each cell line and the presentation of MHC I at the cell surface (Pearson’s r = 0.96). *B*, stacked bar chart displaying the number of unique peptides identified in primary tissue samples, and a horizontal line indicating the average number of MAPs per tissue sample. “All peptides” in (*A* and *B*) indicates the number of peptides identified with a 5% FDR, whereas “MHC I peptides” indicates the number of peptides identified with the corresponding peptide score, 8–11 amino acids in length, and a rank eluted ligand threshold ≤2% using netpanMHC4.1b predictions. *C*, bar chart indicating the proportion of unique MAPs predicted to bind to a given HLA allele in each sample, using NetMHCpan-4.1b predicted affinity. *D*, GO term analysis of MAP source genes for CRC-derived cell lines and primary tissues. For tissues, only source genes shared by four or more tissues were included in this analysis. *E*, *left panel*: Stacked bar chart displaying the proportion of MAPs in each tissue sample derived from protein-coding, hypervariable gene (immunoglobulin or T cell receptor), or noncoding transcripts, or those from unannotated transcripts. *Right panel*: stacked bar chart displaying the proportion of noncoding MAPs derived from processed transcripts, retained introns, nonstop decay products, nonsense mediated decay products, lncRNA, or those that have no annotated transcript. CRC, colorectal cancer; GO, gene ontology; MAP, MHC I–associated peptide.

To elucidate the MHC I immunopeptidomes of CRC-derived cell lines and tissues, we immunoprecipitated MAPs from four replicates of 2 × 10^8^ cells for each cell line and from each tissue sample. We then derivatized each replicate with a separate TMT6plex channel (channels 126, 127, 128, 129) for cell lines or with TMT10plex-126 and -127N for primary NAT and tissue samples, respectively. The four replicates of each cell line, and half of the respective NAT and tumor MAPs from each subject, were multiplexed and analyzed by LC-MS/MS. The median labeling efficiencies were 72.4% or 87.8% for cell lines and tissue samples, respectively. We ascribe the lower efficiency of labeling in cell lines to meager MAP yields. We identified 5281 and 27,583 unique MAPs in the cell line and tissue datasets, respectively, with a mean of 1433 unique MAPs per cell line and 5855 per tissue (Fig. 3*A*, upper panel, and 3*B*, supplemental Files S1 and S2). Although the identification varied between each line, the number of MAPs identified was strongly correlated with the abundance of MHC I molecules per cell (Fig. 3*A*, lower panel; Pearson’s r = 0.96).

When taking the cell line and tissue samples together, we identified a total of 30,485 unique MAPs. Within the MAP repertoire of each sample, 32% to 68% of the peptides are sample specific, and very few shared MAPs were observed when comparing only cell line or primary samples (supplemental Fig. S5, *A* and *B*). This large proportion of unique MAPs can be attributed to the diversity of HLA alleles among our samples, which is a major factor influencing which peptides can be presented at the cell surface (Figs 3*C* and S2). On average, the number of MAPs shared by any two cell lines or any two tissue samples is 59 or 640 MAPs, respectively. There are noteworthy outliers— tissue samples S1 and S6 shared 2079 MAPs (1673 of which are unique to these samples [supplemental Fig. S5*C*]), thus their MHC I immunopeptidomes have approximately 23% similarity, as measured by the Jaccard index. (supplemental Fig. S5*D*). The next closest similarity in MAP repertoires between two tissues is 1328 MAPs shared by the two MSI tissues (S5 and S6), which is only an 11% similarity between their immunopeptidomes. Despite the decreased MAP identification in cell lines, these trends are reproduced. For example, HCT116 and RKO share the most MAPs, although these peptides represent only 4% similarity, and this is likely a feature of their larger peptide repertoires (supplemental Fig. S5*C*). In contrast, COLO205 and SW620 share 152 MAPs, approximately 10% similarity.

To contextualize these comparisons, we can again consider the HLA alleles of our samples. Of the 2079 MAPs shared by S1 and S6, 1595 MAPs are predicted to bind the same allele HLA-B∗07:02 in approximately 90% of these cases (supplemental File S2). In addition, the S1 allele HLA-A∗24:02 and the S6 allele HLA-A∗23:01 have very similar allele-binding motifs as shown by HLAthena (63). Similarly, 126 of 152 MAPs shared by COLO205 and SW620 are bound by the allele HLA-A∗02:01 for 94 MAPs (supplemental File S1). Thus, the MHC I immunopeptidomes of our samples is majorly influenced by the HLA repertoire.

At the gene level, we identified peptides derived from over 8000 unique source genes, with an average of 1014 and 3168 source genes per cell line and tissue sample, respectively (supplemental Fig. S6*A*, upper panel). This was highly correlated with the number of MAPs identified (supplemental Fig. S6*A*, lower panel). Roughly 6% to 14% of the source genes in a given immunopeptidome were sample specific (supplemental Fig. S6*B*), which could be attributed to sample-specific biological features or could reflect an imperfect sampling of the immunopeptidome (supplemental Fig. S6*E*). We do not expect to identify every MAP presented at the cell surface, and as a majority of source genes in each sample are attributable to only a single MAP (supplemental Files S1 and S2), it is almost certain that additional source genes contribute to the MAP repertoire and their corresponding peptides are simply not detected. When comparing any two tissue samples, they had on average 32% source gene similarity, while comparing any two cell lines resulted in an average of 13% shared gene similarity (supplemental Fig. S6, *C*–*E*). Thus, distinct cell lines appear to be less homogenous than tissue samples at the source gene level. This likely reflects differences in sample composition, as the tissue samples have source genes derived from NAT, stroma, infiltrating cells, etc., whereas cell lines consist of only a single cell type. In addition, lower MHC I presentation of cell lines and the resulting decreased identification of MAPs means fewer source genes were sampled, lowering the likelihood of overlap. Regardless, all samples are more similar at the source gene level compared with the immunopeptidome level, and sample-specific MAPs are being derived from shared source genes.

To obtain an overview of the genomic function of the MHC I immunopeptidome and investigate the overlap of source genes, we performed GO term analysis on all the source genes identified in the cell lines as well as those identified in four or more tissues. Several common features between cell lines and tissues are detectable at the immunopeptidome level, including a significant enrichment of genes involved in RNA metabolism, ribonucleoprotein complex biogenesis, translation, and cellular responses to stress (Fig. 3*D*). Thus, despite the large diversity of HLA alleles between and among our cell lines and tissue samples and the low MAP identification in cell lines, there is significant similarity in terms of what genes are contributing to the MHC I immunopeptidome.

To investigate what proportion of MAPs from our tissue samples were from noncoding transcripts, we first determined, for each peptide, the most abundant putative source transcript (Ensembl Annotation 99). For peptides from the cancer-specific database, we mapped the MCS onto the genome and determined the most expressed transcript at that location (see “Quantification of MAP Coding Sequences in RNA-Seq Data” in Methods section). We thus determined that, on average, 95.3% of our MAPs from tissue samples were from protein coding transcripts (*i.e.* UTR or CDR) (Fig. 3*E*, left panel). Approximately 4.2% of peptides are from noncoding regions if we include the 2.8% of peptides deriving from unannotated RNA transcripts, as these peptides are likely coming from intergenic sequences. Approximately one-third of all noncoding MAPs (including those from unannotated transcripts) are derived from nonsense-mediated decay transcript products, whereas less than 1% of them are coming from long noncoding RNA (lncRNA), nonstop decay products, retained introns, or processed transcripts (transcripts that do not contain open reading frames) (Fig. 3*E*, right panel).

## Identification of Tumor-Specific and Tumor-Associated Antigens in CRC

Following the identification of over 30,000 unique MAPs, we filtered peptide-coding sequences to select those overexpressed at least 10-fold in cancer and expressed ≤2KPHM in pooled TEC samples or matched NAT, for cell lines and primary samples, respectively. A recent immunopeptidomic study in acute myeloid leukemia demonstrated that MCSs with RPHM <8.55 have less than 5% probability to generate MAPs (18). We thus quantified the expression of the MCSs in RNA-Seq data and kept only those that were expressed below 8.55 RPHM in mTECs and other normal tissues (GTEx). Following manual validation of the remaining peptides, we classified peptides as mutated TSAs (mTSAs) if their amino acid sequence contained a cancer-specific mutation (*i.e.* not an SNP). MAPs for which the sequence was the same as the reference genome and overexpressed at least 10-fold in tumor compared with normal were classified as aberrantly expressed TSAs (aeTSAs) if they had no or residual RNA expression (≤0.2 KPHM) in mTECs (and NAT in the case of tissues) or as TAAs if their expression in mTEC and/or NAT was greater than 0.2 KPHM.

**Fig. 4:**
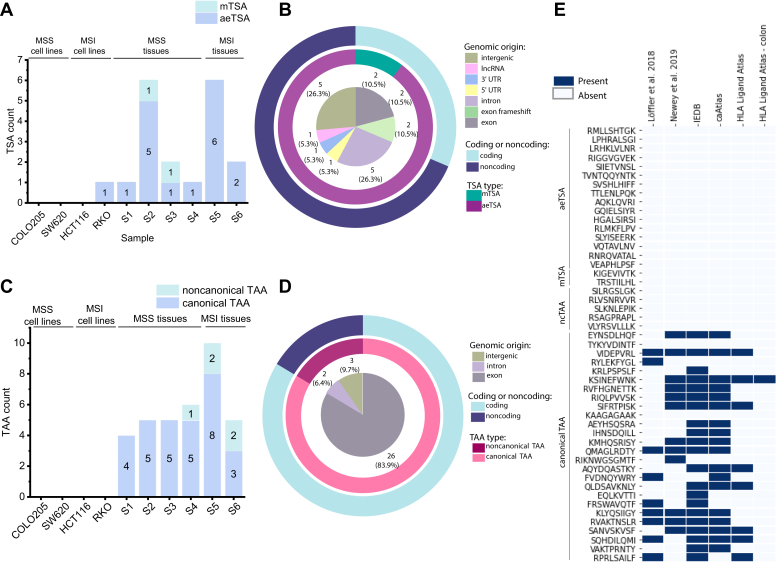
**Novel TSAs identified in colorectal cancer derive primarily from noncoding regions, whereas the majority of TAAs derive from exons**. *A*, bar chart displaying the number of TSAs identified per sample. *B*, stacked pie chart identifying the genomic origin of TSAs in the inner pie, as well as what proportion of TSAs are mutated in the middle pie. The outer pie demonstrates what proportion of TSAs are from coding or noncoding sequences. *C*, bar chart displaying the number of TAAs identified per sample. *D*, stacked pie chart identifying the genomic origin of TAAs in the inner pie, and what proportion of TAAs are canonical or noncanonical in the middle pie. The outer pie displays what proportion of TAAs are from coding or noncoding sequences. *E*, heatmap displaying the presence or absence of putative TSAs and TAAs in two previous publications on CRC immunopeptidomics (Löffler *et al.* 2018 and Newey *et al.* 2019), as well as IEDB and HLA Ligand Atlas (all tissues, and only colon tissue). MSI, microsatellite instability; MSS, microsatellite stable; TAA, tumor-associated antigen; TSA, tumor-specific antigen.

Although the TSA yield in CRC-derived cell lines was relatively meager, possibly due in part to low MAP identification, we uncovered an average of three TSAs per primary tissue sample (Fig. 4*A*). Overall, we identified 1 putative TSA in a CRC-derived cell line and 18 putative TSAs in primary tissues, and the TSA yield from each sample was correlated with the number of MAPs identified (Pearson’s r = 0.76) (supplemental Fig. S7). Of these, approximately one-third were derived from coding regions, whereas the majority of the putative TSAs identified originated from noncoding regions (Fig. 4*B*). Among the TSAs from coding regions, two were from noncanonical reading frames, deriving from exon frameshift sequences, and another two were mutated TSAs identified in MSS tissues S2 and S3 (Fig. 4, *A* and *B*). Among the noncoding TSAs, a large proportion originated from intronic or intergenic regions, with a smaller number being derived from 5′ UTR, 3′ UTR, or lncRNAs (Fig. 4*B*). The sequences of six aeTSAs (four introns, one intergenic, one lncRNA) overlapped endogenous retroviral element (ERE) sequences (supplemental File S3). Owing to the ubiquitous nature of EREs, TSAs derived from aberrant ERE expression are potentially shared by tumors and have been shown to be immunogenic (64, 65). Of note, none of our putative TSAs were shared between samples, even those with a high proportion of shared MAPs. However, we did identify two unique TSAs in different tissues that were derived from the same transcript of COL11A1 (one exon frameshift and one 5′ UTR), which was recently shown to play a role in CRC development and prognosis (66). The majority of other TSA source genes have also been shown to be biologically relevant in CRC (Table 3).

**Table 3:** Biological relevance of TSA source genes in CRC

Source gene	Reference	Biological relevance in CRC
COL11A1—Collagen type XI alpha 1	PMID: 33597969	Upregulated in CRC (mRNA), marker of poor prognosis, role in CRC development
CYP39A1—cytochrome P450, family 39, subfamily A, polypeptide 1	PMID: 27341022	Expression is increased in CRC with poor prognosis
DPH6—Diphthamine biosynthesis 6		No known association
GRIN2B—Glutamate ionotropic receptor NMDA type subunit 2B	PMID: 27243824	Identified as nondriver hub gene involved in progression to stage II CRC
HKDC1—Hexokinase domain-containing protein 1	PMID: 30005951	HKDC1 contributes to increased metabolism, proliferation, and metastasis of CRC cells
HSPD1—Heat shock protein family D (Hsp60) member 1	PMID: 28261350; PMID: 29246022	Differentially expressed in CRC, potential biomarker for diagnosis; exosomal HSPD1 identified as putative diagnostic and prognostic biomarker in CRC
IPP (KLHL27)—Intracisternal A particle-promoted polypeptide	Human Protein Atlas (PMID: 28818916)	Favorable prognostic marker in colorectal cancer, unfavorable in renal and liver cancers
LY6G6F-LY6G6D readthrough—Lymphocyte antigen 6 family member G6F and G6D	PMID: 26894861	LY6G6D/F overexpressed in CRC, potential cell surface marker
NKD1—Naked cuticle homolog 1	PMID: 25446263; PMID: 19956716	Negative feedback regulator of Wnt pathway, intestinal tumor marker in mice; mutations in NKD1 alter Wnt signaling
PATJ—PALS1-associated tight junction protein		No known association
PLK1—Serine/threonine-protein kinase PLK1/polo-like kinase 1	PMID: 22648245	Overexpressed in CRC, associated with metastasis and invasion
SUCNR1—Succinate receptor 1	PMID: 32365557	SUCNR1 activation induces Wnt ligand expression and activates WNT signaling and EMT in a CRC-derived cell line
TRPC6—Transient receptor potential cation channel subfamily C member 6	PMID: 26422106	mRNA expression of TRPC6 lower in CRC than in normal tissue, may contribute to tumorigenesis

Although our primary objective was to identify putative TSAs in CRC, we also identified an average of 5.2 TAAs in our CRC tissue samples, although none were identified in our CRC-derived cell lines (Fig. 4*C*). In contrast to the primarily noncoding putative TSAs, the majority of the TAAs we identified were from canonical, exon-coding sequences, with only a small number being derived from introns or intergenic sequences (Fig. 4*D*). Two noncanonical TAAs overlapped ERE sequences (supplemental File S3). Of note, four separate TAAs were identified in more than one sample. These shared TAAs were all derived from canonical exons, with source transcripts originating from ASPM, MKI67, MMP12, and HI-5, all of which have documented associations with cancer (Table 4).

**Table 4:** Biological relevance of TAA source genes in CRC

Source gene	Reference	Biological relevance in CRC
**ASPM—Abnormal spindle microcephaly associated**	**PMID:**31966766**; Human Protein Atlas (PMID:**28818916**)**	**Overexpressed in CRC; suggested to be unfavorable prognostic marker (involved in mitosis, cell cycle, tumorigenesis); known to be unfavorable prognostic marker in liver, lung, endometrial, pancreatic cancers**
BUB1—Mitotic spindle checkpoint kinase	PMID: 23747338; PMID: 11782350	Mutations in BUB1 linked to early onset CRC; inactivation may drive metastasis and progression in CRC
CDCA8—Cell division cycle associated 8	PMID: 25260804	Overexpressed in CRC, associated with cancer progression
CENPE—Centromere-associated protein E		No known association
**CENPF—Centromere protein F**	**PMID:**30550624	**Phosphorylation changes associated w CRC malignancy; unfavorable prognostic marker in other cancers (liver, renal, etc.; human protein atlas)**
DIAPH3—Diaphanous related formin 3	Human Protein Atlas (PMID: 28818916)	DIAPH3 is prognostic, high expression is favorable in colorectal cancer
**FANCA**—**Fanconi anemia group A protein**	**PMID:**27165003**; PMID:**21286667	**Fanconi anemia predisposes certain cancers; genes in FA pathway participate in CRC pathogenesis (involved in HR repair)**
HI-5—H1.5 linker histone, cluster member	PMID: 16959974	Frequently mutated in CRC
IDO2—Indoleamine 2,3-dioxygenase 2	PMID: 18418598	Upregulated expression in CRC
MACC1—Metastasis-associated in colon cancer 1	PMID: 27424982; PMID: 25003996	Promotes growth and metastasis of colorectal cancer; associated with carcinogenesis through B-catenin signaling and EMT transition
MCM10—Minichromosome maintenance 10 replication initiation factor	PMID: 32597491	Decreased mRNA expression in colon and rectal adenocarcinoma samples compared with normal tissues
MGAM2—Maltase glucoamylase 2	PMID: 30996822	Expressed in GI cancers (TCGA data)
**MKI67—Marker of proliferation Ki-67**	**PMID:**26281861**; PMID:**27855388**;** **PMID:**30727976**; PMID:**33658388	**Favorable prognostic marker in CRC, IHC staining (2016); favorable prognostic marker in stage III and IV CRC, IHC staining (2016); poor prognostic marker in CRC based on database meta-analysis (2019); Ki-67 expression important for tumorigenesis**
**MMP12**—**Matrix metallopeptidase 12**	**PMID:**27431388	**Overexpressed in CRC compa**red with **control, negative prognostic marker in CRC**
NOS2—Nitric oxide synthase 2	Human Protein Atlas (PMID: 28818916)	Cancer enhanced (colorectal cancer); RNA data
**SPC25 (kinetochore protein)**	**PMID:**32351050**; Human Protein Atlas (PMID:**28818916**)**	**Highly expressed in CRC (among other cancers); unfavorable prognostic marker in liver cancer, endometrial cancer, and lung cancer**
ZNF215—Zinc finger protein 215	Human Protein Atlas (PMID: 28818916)	Cytoplasmic expression in subsets of immune cells, most abundant in gastrointestinal tract and lymphoid tissues (protein data)

Bold, validated.

We initially expected to identify an above-average number of both TSAs and TAAs in MSI tissues. This was the case in S5; however, the same was not true for the other MSI tissue (Fig. 4, *A* and *C*). This could be due to S6 having a lower “degree” of instability, as reflected in the MSIsensor-pro results (supplemental Table S1). Furthermore, the sample that had the highest number of identified TSAs was S2, an MSS tissue. Thus, the yield of TSAs and TAAs per sample seems to be irrespective of MSI status and may be due to other unique biological features of the tumor outside the scope of this study.

To determine if any of our putative TSAs or TAAs have been previously identified, we verified if the peptide sequences were reported in the Immune Epitope Database, caAtlas (67), the HLA Ligand Atlas (68), and two previous publications that sought to identify tumor antigens in CRC from Löffler *et al.* 2018 (8) and Newey *et al.* 2019 (15). Of note, none of the putative aeTSAs, mTSAs, or noncanonical TAAs were previously reported in any of these resources. Of the 26 putative canonical TAAs identified, 24 of them were reported either in the Immune Epitope Database (IEDB), caAtlas, Löffler *et al.* 2018, Newey *et al.* 2019, or some combination of the four (Fig. 4*E*). Eight of these were also reported in the HLA Ligand Atlas, with one of them specifically being documented in healthy colon tissue. Interesting, none of the TAAs previously identified in these earlier publications were reported as tumor antigens, and, conversely, six of the 12 tumor antigens of interest reported in Loffler *et al.* were also identified in the immunopeptidomes of our work, although they did not pass our TSA or TAA selection criteria, most often due to high expression in NAT (supplemental Table S2). We have thus identified novel TSAs in CRC that derive primarily from noncoding regions, as well as a selection of mainly coding TAAs, some of which have been previously reported as MAPs.

## RNA Expression Of Putative Tumor-Specific and Tumor-Associated Antigens

**Fig. 5:**
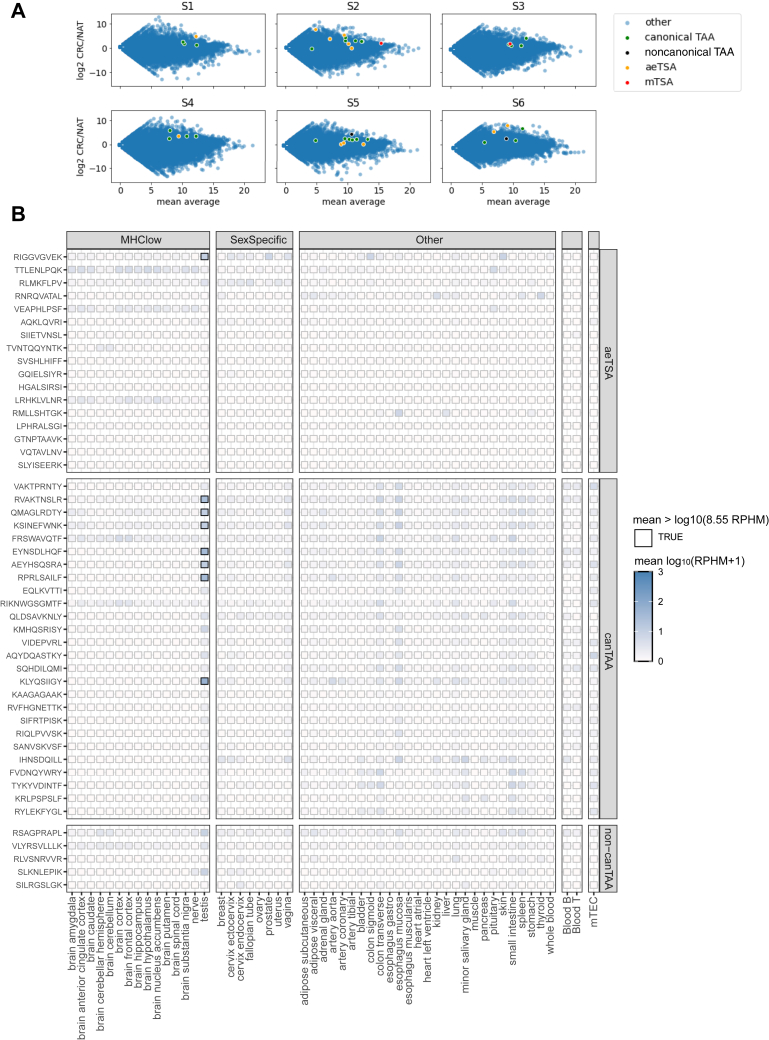
**RNA expression profiles of putative TSAs and TAAs**. *A*, scatter plots displaying the log2FC of transcripts, in TPM, in CRC compared with the matched NAT on the *y*-axis and the mean average expression in a given tissue sample (mean of CRC and NAT). Highlighted points indicate the source transcripts of putative TAA and TSAs. Both S4 and S5 plots have a canonical TAA point that is not visible, as it overlaps with another canonical TAA source transcript. *B*, heatmap of mean RNA expression in log(rphm+1) of aeTSA coding sequences and TAA coding sequences (divided as canonical TAAs [canTAA] and noncanonical TAAs [non-canTAA]) in normal tissues from Genotype Tissue Expression (GTEx) Portal and in pooled thymic epithelial cell samples. MHClow tissues include those from brain, nerve, and testis, which have been shown to lowly express MHC I. A *black* outline indicates a mean RNA expression >8.55 rphm. aeTSA, aberrantly expressed tumor-specific antigen; CRC, colorectal cancer; NAT, normal adjacent tissue; TAA, tumor-associated antigen; TSA, tumor-specific antigen.

First, we investigated the expression, in TPM, of the source transcripts in their respective tumor samples compared with the matched NAT, as well as the mean average of that transcript in the CRC/NAT sample (Fig. 5*A*). This analysis naturally does not include peptides derived from intergenic regions. Note that, in Figure 5*A*, both S4 and S5 plots have a canonical TAA point that is not visible, as it overlaps with another canonical TAA source transcript; however, these sequences were still included in downstream analyses. The average log2FC for the source transcripts of our putative TSAs and TAAs in the samples in which they were identified was 3.6 and 3.2, respectively. In some instances, the source transcript of an aeTSA was only slightly more abundant in the tumor than in the NAT; however, this reflects only the overall abundance of the entire transcript, and the peptide-coding sequences were in fact more abundant in the cancer (supplemental Fig. S8). This was also true for aeTSAs, in which the peptide-coding region was either entirely absent or lowly expressed in the NAT but was more highly expressed in the cancer tissue.

To evaluate the specificity of our putative tumor antigens, we determined the mean expression of the peptide-coding sequences in the large dataset of healthy tissues provided by the Genotype-Tissue Expression project (GTEx) (Fig. 5*B*). The TSA sequences were not expressed above 8.55 RPHM in any healthy tissues, except RIGGVGVEK, an aeTSA identified in S2, which was expressed above threshold in the testis. This suggests that this TSA could also be classified as a cancer-testis antigen (CTA), a class of aeTSA that is expressed in male germ cells but may also be aberrantly expressed in cancer. Owing to the absence of MHC I in testis, these antigens are also promising candidates for cancer immunotherapy (69). This putative TSA is an LY6G6F-LY6G6D exon frameshift. Although these genes have not been previously reported as CTAs, another member of the same gene family, LY6K, has been reported as a CTA in lung and esophageal cancers (70). TAA expression was below threshold in healthy tissues, although it tended to be higher in the esophagus and the transverse colon. Seven of these peptides were also expressed above threshold in the testis.

## Cancer Specificity and Immunogenicity Prediction of TSAs and TAAs

Following our identification of putative TSAs and TAAs, we validated all of the TSAs and a subset of nine TAAs with synthetic peptides. These TAAs were selected based on favorable initial TMT intensity ratios and precursor ion fractions in cancer *versus* matched NAT. These candidates all had MS/MS that correlated well with those of the synthetic peptides, with Pearson correlation score ≥0.6 (supplemental Fig. S9). We then labeled synthetic peptides with TMT10plex-129N,130N, and 131 at concentrations of 10, 100, and 1000 fmol, respectively, and spiked into remaining purified MAPs from tissue samples that were labeled with TMT126 (NAT) and 127N (CRC). SPS-MS3 was then used to quantify peptides of interest in these samples. Despite the decreased sensitivity of SPS-MS3, we were able to quantify seven TSAs and seven TAAs. We selected good-quality PSMs for quantification, and as expected for antigens of this nature, all were more abundant in their respective CRC compared with NAT (Table 5). Determining the ratio of intensity of TMT127N peptides compared with TMT126 peptides revealed that TSAs had a median intensity fold change of 16.96 in CRC compared with NAT, whereas TAAs had a fold change of 6.93. In addition, the TSA with sequence RYLEKFYGL was also overexpressed in the S1 tumor, despite only passing our transcriptomic thresholds for S6. Thus, we were able to demonstrate that the TSA identification methodology used in this study successfully identified TSA and TAA sequences that are more highly abundant at the surface of cancer cells than that of NAT.

**Table 5:** Relative quantification ratios of validated tumor antigens in CRC

Sequence	Nature of antigen	Sample	Endogenous sample ratio	Mean intensity	SPS-MS3 ratio (127N/126)	Synthetic calibration curve R^2^
RMLLSHTGK	aeTSA	RKO	N.D.	N.D.	N.D.	N.D.
LPHRALSGI	aeTSA	S1	−0.364	N.D.	N.D.	N.D.
GTNPTAAVK	aeTSA	S2	2.095	7238.425242	12.174	1.000
LRHKLVLNR	aeTSA	S2	0.307	N.D.	N.D.	N.D.
RIGGVGVEK	aeTSA	S2	1.965	29256.45	6.740	1.000
SIIETVNSL	aeTSA	S2	0.288	N.D.	N.D.	N.D.
TVNTQQYNTK	aeTSA	S2	−0.021	N.D.	N.D.	N.D.
SVSHLHIFF	aeTSA	S3	−1.100	N.D.	N.D.	N.D.
TTLENLPQK	aeTSA	S4	0.134	3140.8875	3.783	0.999
AQKLQVRI	aeTSA	S5	0.793	N.D.	N.D.	N.D.
GQIELSIYR	aeTSA	S5	0.328	N.D.	N.D.	N.D.
HGALSIRSI	aeTSA	S5	0.777	N.D.	N.D.	N.D.
RLMKFLPV	aeTSA	S5	0.171	N.D.	N.D.	N.D.
SLYISEERK	aeTSA	S5	0.046	N.D.	N.D.	N.D.
VQTAVLNV	aeTSA	S5	1.089	N.D.	N.D.	N.D.
VEAPHLPSF	aeTSA	S6	1.059	43782.84192	41.318	1.000
RNRQVATAL	aeTSA	S6	1.090	12174.6625	5.722	1.000
RNRQVATAL	Not assigned	S1	0.890	15514.2375	3.507	1.000
KIGEVIVTK	mTSA	S2	2.506	70659.6	13.637	1.000
TRSTIILHL	mTSA	S3	1.381	34365.32187	48.807	0.997
VLYRSVLLLK	Noncanonical TAA	S6	0.997	N.D.	N.D.	N.D.
TYKYVDINTF	Canonical TAA	S1	1.969	29834.36875	8.226	0.998
RYLEKFYGL	Canonical TAA	S1	2.840	27614.24286	7.661	0.997
RYLEKFYGL	Canonical TAA	S6	2.970	106928.2875	16.090	0.999
KSINEFWNK	Canonical TAA	S2	2.212	56110.11667	5.238	0.999
RIQLPVVSK	Canonical TAA	S4	1.083	7612.378571	2.073	0.999
QMAGLRDTY	Canonical TAA	S3	1.140	36090.60294	2.884	0.999
AQYDQASTKY	Canonical TAA	S4	1.452	N.D.	N.D.	N.D.
FVDNQYWRY	Canonical TAA	S4	0.721	5853.986533	10.954	1.000
SANVSKVSF	Canonical TAA	S5	1.114	12780.925	2.321	0.999

N.D.: not detected.

Endogenous sample ratio: 127N/126 ratio in endogenous samples.

**Fig. 6:**
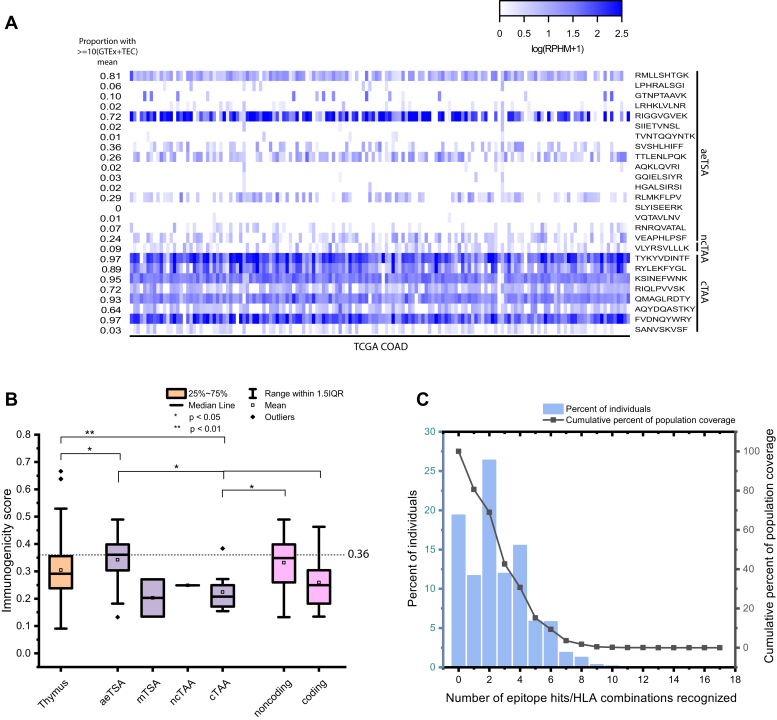
**Validation of TSAs and TAAs**. *A*, heatmap displaying mean RNA expression in log(rphm+1) of TSAs and TAAs in 151 TCGA COAD samples. The proportion of TCGA COAD samples expressing the TSA and TAA sequences at least 10-fold higher than the log-transformed (log(rphm+1)) mean expression of pooled GTEx and mTEC samples is displayed on the *left*. *B*, rEpitope immunogenicity scores of various groupings of validated TSAs and TAAs compared with presumably nonimmunogenic thymic peptides reported in Adamopoulou *et al*. 2013. rEpitope suggested threshold of immunogenicity for MHC I peptides (0.36) is indicated by the *dashed line*. *C*, predicted prevalence of tumor antigen-binding MHC class I alleles in US population (IEDB). COAD, colon adenocarcinoma; GTEx, Genotype Tissue Expression project; mTEC, medullary thymic epithelial cell; TAA, tumor-associated antigen; TSA, tumor-specific antigen.

To examine the intertumoral distribution of these TSAs and TAAs in other CRC tumors, we plotted the log(RPHM+1) expression of the peptide coding sequences in 151 colon adenocarcinoma samples from The Cancer Genome Atlas (TCGA) (Fig. 6*A*). To evaluate the sharing potential of our antigens, for each peptide of interest, we first calculated the average of log-transformed (log(rphm+1)) values of pooled GTEx (n = 2442) and mTEC (n = 8) samples. Overall, nine TSAs (53%) and nine TAAs (100%) had an expression ≥10-fold above their corresponding averaged GTEx/mTEC value in at least 5% of TCGA COAD tumors. This demonstrates that TAAs are more frequently shared among COAD TCGA tumors than their TSA counterparts. However, this also means that most TSAs are highly shared in these samples.

Another important consideration in the identification of tumor antigens is whether these peptides are able to invoke an effective antitumor immune response. Repitope predictions of immunogenicity revealed that our aeTSAs are predicted to be significantly more immunogenic than a set of thymic peptides, which are presumed nonimmunogenic (71) (Fig. 6*B*). In addition, aeTSAs had significantly higher immunogenicity scores compared with canonical TAAs and coding TAs overall (TSAs and TAAs derived from coding regions). In fact, TAAs from canonical regions were predicted to be significantly less immunogenic than thymic peptides (*p* < 0.01). This could be partially due to the low number of TAAs that we validated. If we consider these predictions with the entire set of 31 TAAs, this is no longer the case (supplemental Fig. S10). Considering all 31 TAAs revealed that MSI TAs are predicted to be more immunogenic than thymic peptides, while there is also a statistically significant increase in predicted immunogenicity of TAs derived from MSI tissues compared with MSS.

Finally, we sought an approximation of the proportion of individuals who possess the alleles that are predicted to bind and present our tumor antigens (Fig. 6*C*). Many of the antigens in our samples are prevalent, and an estimation with the IEDB population coverage tool predicted that 80.64% of the US population expresses at least one of the alleles associated with the TAs identified in this study.

## Discussion

Mass spectrometry is currently the best method to identify MAPs of interest, as it can directly sample the MHC I immunopeptidome and eliminates the need for error-prone prediction software, which are unable to incorporate the largely misunderstood intricacies of MAP processing and presentation (72). Our approach has previously led to the identification of TSAs in lung cancer (16), ovarian cancer (17), and acute myeloid leukemia (18), the majority of which are aberrantly expressed and derive from noncoding regions. The addition of TMT labeling allows us to both improve MAP identification and quantify peptide abundance between samples (40). Although TSAs and TAAs have been identified in CRC, studies to date have only taken interest in the coding portion of the genome. By elucidating the MHC I immunopeptidomes derived from both canonical and noncanonical sequences in CRC cell lines and tumors, we thus present, to our knowledge, the first successful identification of aberrantly expressed TSAs in CRC. As novelty to our well-established identification workflow, we incorporated matched NAT of the respective CRC primary samples in our analysis, thus allowing for the most accurate possible “control” samples of normal expression of peptide-coding regions. The aeTSAs identified here derive primarily from noncoding regions, which has also been previously demonstrated in other cancers (16, 17, 18).

MSI tumors are characterized by increased immune infiltration (Figs. 2*C* and S3*B*) and more favorable responses to ICI (particularly PD-1 inhibition) compared with their MSS counterparts (5, 73). The increased mutational load (Figs. 2*E* and S4, *B* and *C*) and increased immunogenicity of MSI in CRC suggested that these tumors would be characterized by a larger TSA or TAA burden. Although the MSI tissue samples were sources of many TSAs and TAAs, we were able to identify 8 aeTSAs, 2 mTSAs, and 18 unique TAAs in MSS tissues (7 of which were validated with synthetic peptides). Thus, it could be that the unfavorable response of MSS tumors to ICI is not due to a lack of tumor antigens but rather to a lack of immune activation against these antigens. Accordingly, when considering all 31 of our identified TAA sequences, we saw a statistically significant decrease in the Repitope immunogenicity scores of TAs derived from MSS tissues compared with their MSI counterparts (supplemental Fig. S10). Although this trend was not observable when only considering our validated TAA sequences, this could be attributed to the decrease in sample size for both subtypes. Despite a population-level decrease in immunogenicity, there are MSS-derived TAs with immunogenicity scores above the suggested threshold, which could still hold promise for immunotherapy. The “immune cold” status of MSS tumors could alternatively be resulting from a lack of recruitment to the tumor site (74). Fortunately, there is a wide array of strategies designed to overcome the lack of immune infiltration into cold tumors, which could perhaps be used in combination with ICI or other immunotherapeutic approaches, such as vaccines, making use of TSAs such as those described here.

Among these TSAs, we identified two mTSAs unique to tumors derived from PLK1 and HDSP1, with missense mutations A520T and V345I occurring in 27% and 49% of RNA-Seq reads, respectively. The HDSP1 mutation is predicted to be benign by software such as Polyphen and CADD (75, 76). Although the PLK1 mutation is documented in dbSNP (rs1004523813), it was not excluded from our analyses as the mutation is tumor specific (not present in paired NAT) and is very rare in the population (minor allele frequency <0.01) (77). Furthermore, it is well documented in COSMIC (cancer.sanger.ac.uk) and is predicted to be pathogenic by the Functional Analysis through Hidden Markov Models (75, 78). A recent study in immunopeptidomics of CRC organoids previously reported the discovery of three mTSAs (15). Unless derived from driver mutations, mTSAs are rare and thus it is unlikely that they are shared between tumors, and these particular mTSAs are absent from our study. In addition to mutation rarity, differences in peptide processing and presentation at least partly attributable to HLA diversity among samples further lessens the likelihood of identifying shared mTSAs. However, it is worth mentioning that MAPs from the source genes were reported in several of our samples (two, three, and three unique peptides from U2SURP, MED25, and FMO5, respectively). When taken together, these observations suggest that the overlap of mTSAs between specimens is relatively low and it is thus not surprising to note the absence of previously reported mTSAs in our study, despite the relatively high SNV burden among our samples (Figs 1*E* and S4*C*). mTSAs are immunologically relevant and have the capacity to be immunogenic, but owing to their lack of sharing between individuals, their potential for use in large-scale immunotherapy is limited.

Although mTSAs are expected to be rare and unique to a given tumor, it has been shown that aeTSAs can be shared among patients (79). Here, we did not identify any common aeTSAs among our six patients; however, we did identify two unique aeTSAs in different patients that were derived from the same transcript of the COL11A1 gene, which is known to be associated with CRC (Table 3). It should be noted that, in this study, we worked with only six primary samples, which were largely diverse in their HLA alleles, thus reducing the likelihood of shared TSAs. The fact that we were still able to identify TSAs from the same transcript is encouraging, as it suggests that this transcript is generating biologically relevant peptides across different tumors. It is possible that these TSAs could be presented by other tumors with similar alleles, or that this transcript could be generating novel TSAs capable of being presented by other HLA alleles not examined here. In addition, the TSA sequence RNRQVATAL was originally identified as a TSA candidate in S6 only. It was not considered a TSA in S1 originally owing to the level of expression in normal tissue (RNA coding sequences not expressed at least 10-fold higher in cancer than in NAT), and yet at the immunopeptidomic level it had a 3.5-fold higher intensity in CRC than in NAT. The fact that this peptide could also be considered a tumor antigen in S1 relates to the fact that mRNA abundance and protein abundance are not highly correlated (80), and our stringent identification pipeline excluded it based on RNA-Seq data. This reinforces the need for mass spectrometry to directly sample the immunopeptidome, to relatively quantify the abundance of such peptides at the cell surface, and to validate the immunogenicity of TSAs in large-scale *in vitro* studies.

Outside of our six tissue samples, the decreased sharing of some TSAs among TCGA COAD tumors suggests that certain TSA sequences are not widely shared (Fig. 6*A*). Of the nine TSAs that are expressed ≥10-fold above their corresponding averaged GTEx/mTEC value in at least 5% of TCGA COAD tumors, three are from intergenic sequences, two from exons, two from exon frameshifts, and one each from intronic or 5′ UTR sequences. This small sample size prevents us from drawing any conclusions; however, there may be a therapeutic advantage to distinguishing highly shared TSAs from those that are less abundant across COAD populations. Although high tumoral RNA expression of a TSA sequence does not guarantee MHC I presentation of that peptide, it does increase the likelihood that a given TA sequence, or perhaps other sequences from the same transcript, could have dysregulated MHC I presentation in cancer.

In contrast to TSAs, multiple canonical TAAs are shared between different primary CRC samples, with up to three samples presenting the same TAA. In addition, the same genes can generate multiple relevant TAAs across tumor samples, with three unique TAAs being derived not only from the ASPM gene but from the same transcript (among these, SANVSKVSF was validated) (Table 4 and supplemental File S3). The increased intertumoral sharing of TAA sequences compared with TSA sequences is also reflected in TCGA data, in which canonical TAA coding sequences are expressed more frequently and more abundantly in colon adenocarcinoma samples compared with their TSA counterparts (Fig. 6*A*). This is to be expected owing to the very nature of TAAs, which are expressed in normal tissues but overexpressed in cases of malignancy, compared with TSAs, which arise only in cases of mutated or aberrantly expressed sequences. As such, TAAs are more challenging to use in immunotherapy approaches as they have been known to induce autoimmune responses, or even T cell tolerance (81). We also demonstrated that the TAAs identified here are predicted to be significantly less immunogenic than the TSAs (Fig. 6*B*). However, TAAs can certainly be advantageous as cancer biomarkers, as is the case with CEA, the first TAA discovered in CRC in the mid-1960s (82).

We were initially surprised at the lack of CEA-derived TAAs in our tissue samples, despite the presence of several CEA-derived MAPs in our dataset (supplemental Files S1 and S2). A closer examination revealed that CEA-derived MAPs (for example, those derived from CEACAM5 or CEACAM7) were excluded from our analysis following the initial peptide classification, which removes MAPs that are not overexpressed at least 10-fold higher in cancer than in matched NAT, and those that are expressed more than 2 RPHM in NAT. In the interest of comparing our findings with other contemporary studies on CRC immunopeptidomics, we queried our dataset for the MAPs identified as potential vaccine candidates in Löffler *et al.* 2018 (8). Of the 12 TAAs they selected, 6 were identified in our tissue samples. However, five of these peptides did not pass the initial classifications in our pipeline (≥10 FC in cancer compared with NAT and ≤2 RPHM in NAT), and the other was found to be expressed more than 8.55 RPHM in mTECs (supplemental Table S2). We would like to note here that our pipeline was designed to identify TSAs and thus has a stringent set of criteria meant to exclude peptides present in normal tissues. As no universal thresholds have been established to classify TAAs, differences in thresholds and filtering steps between studies will naturally result in differential TAA identification. Löffler *et al.* also demonstrated T cell responses to their TAAs (8), suggesting that these antigens do have clinical potential.

Although this study is not meant to be a comprehensive view of CRC immunopeptidomics, the primary goal of our work was to provide a proof of concept that aeTSAs can be identified and are more abundantly presented at the cell surface of CRC than of paired NAT. Despite typical limitations of immunopeptidomic studies such as the amount of available material (particularly for tissue biopsies) and instrument sensitivity, we present here the identification of 19 TSAs. An additional drawback of this study was the low MAP identification in CRC cell lines attributable to the low MHC I abundance at the cell surface, which decreased the probability of identifying TSAs. In the future, MHC I presentation of cell lines could be boosted with IFN-γ treatment to increase identification, particularly of lowly abundant peptides (83). Future investigations will include evaluations of TSA and TAA immunogenicity with T cell assays. In addition, expanding the sample size with primary tissues sharing common HLA alleles could drastically increase the likelihood of identifying shared TSAs. Here, we examine only primary samples of stage 2 nonmetastatic CRC. Differential peptide presentation could be occurring at other tumor stages due to alterations in tumor biology. Expanding the reach of this study could include a large-scale analysis of multiple CRC samples as well as the investigation of TSAs in other stages of CRC.

## Data Availability

The mass spectrometry proteomics data have been deposited to the ProteomeXchange Consortium (84) *via* the PRIDE partner repository (85) with the dataset identifier PXD028309 and 10.6019/PXD028309.

The transcriptomic data discussed in this publication have been deposited in NCBI's Gene Expression Omnibus (86) and are accessible through GEO Series accession number GSE195985 (https://www.ncbi.nlm.nih.gov/geo/query/acc.cgi?acc= GSE195985).

## Supplemental data

This article contains supplemental data.

## Conflict of interest

J. C., M.-P. H., P. T., and C. P. are named inventors on a patent application filed by Université de Montréal and covering antigens described in this article.
