# Supplementary material for: Immunopeptidomic analyses of colorectal cancers with and without microsatellite instability
Source: Mol Cell Proteomics. 2022 Apr 1;21(5):100228. doi: 10.1016/j.mcpro.2022.100228 (PMC9134101; doi:10.1016/j.mcpro.2022.100228)
Supplement: Supplemental Tables S1 and S2, Figures S1–S10 [file mmc4.pdf]

Title: Immunopeptidomic analyses of colorectal cancers with and without microsatellite instability

Authors: Jenna Cleyle[1,2], Marie-Pierre Hardy[1], Robin Minati[1,2], Mathieu Courcelles[1], Chantal Durette[1], Joel Lanoix[1], Jean-Philippe Laverdure[1], Krystel Vincent[1], Claude Perreault[1,3], Pierre Thibault[1, 4.]

Keywords: colorectal cancer, tumor-specific antigen, cancer immunotherapy, immunopeptidomics, mass spectrometry

[1] Institute for Research in Immunology and Cancer, Université de Montréal, Montreal, QC, Canada. [2] Molecular Biology Program, Université de Montréal, Montreal, QC, Canada. [3] Department of Medicine, Université de Montréal, Montreal, QC, Canada. [4] Department of Chemistry, Université de Montréal, Montreal, QC, Canada.

Running Title: Discovery of tumor-specific antigens in colorectal cancer

Corresponding authors: Dr. Pierre Thibault, Institute for Research in Immunology and Cancer, PO Box 6128, Downtown Station, Montreal, QC, H3C 3J7, Canada. Phone: 514-343-6910; Fax: 514-343-6843; E-mail: pierre.thibault@umontreal.ca and Claude Perreault, Institute for Research in Immunology and Cancer, P.O. Box 6128, Downtown Station Montreal, Québec H3C 3J7 CANADA. Phone: 514-343-6126; Fax: 514-343-5839; E-mail: claude.perreault@umontreal.ca

Supplementary Tables

Supplementary Table 1. MSISensor-pro results for CRC primary tissues.

Sample	Number of total sites	Sites with enough coverage	Sites with enough coverage (%)	MSI sites (somatic)	MSI sites (somatic) (%)	Class(MSI > 3.5%)
**S1**	1011195	111243	11	226	0.20	MSS
**S2**	1011195	69004	7	197	0.29	MSS
**S3**	1011195	56779	6	104	0.18	MSS
**S4**	1011195	45848	5	177	0.39	MSS
**S5**	1011195	78340	8	8267	10.55	MSI
**S6**	1011195	60715	6	3085	4.08	MSI

Supplementary Table 2. Justifications for exclusion of Loffler *et al.* 2018 tumor antigens.

Peptide	Loffler et al. 2018 classification	Loffler et al. 2018 HLA restriction	Sample	Reason for exclusion
**RLASRPLLL**	Vaccine candidate	B*07	S5	FC < 10 between cancer and NAT
**YRNSYEIEY**	Vaccine candidate	C*07	S1, S2, S6	MCS expression > 2 KPHM in NAT
**APTPARPVL**	Vaccine candidate requiring further validation	B*07	S1	MCS expression > 8.55 RPHM in mTEC
**RLAEPSQMLK**	Vaccine candidate requiring further validation	A*03	S6	MCS expression > 2 KPHM in NAT
**SPKATGVFTTL**	Vaccine candidate requiring further validation	B*07	S1, S6	MCS expression > 2 KPHM in NAT
**SVLTQPPSV**	Vaccine candidate of unclear relevance	A*02	S2	MCS expression > 2 KPHM in NAT

Supplementary Figures

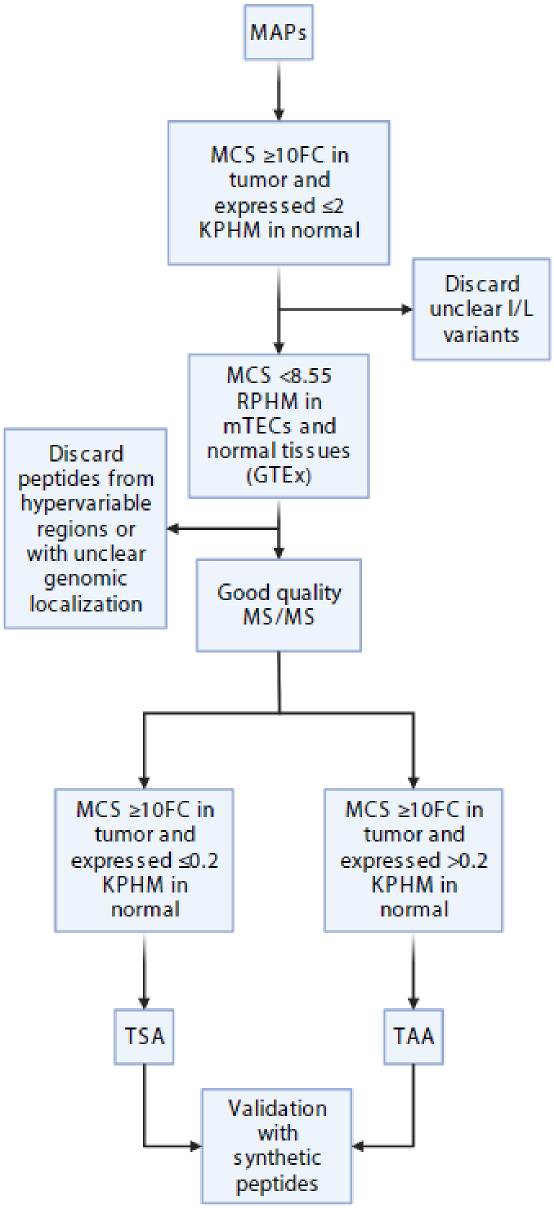

Supplementary figure S1. Flowchart depicting workflow and filters used to identify TSA and TAA candidates, as described in methods. MHC I-associated peptides were selected based on the overexpression of their RNA coding sequences in tumor compared to normal, and their low expression in mTECs and normal tissues. Peptides with from hypervariable regions or with unclear I/L variants or genomic localizations were discarded. Putative TSAs and TAAs were distinguished based on their expression in normal tissues.

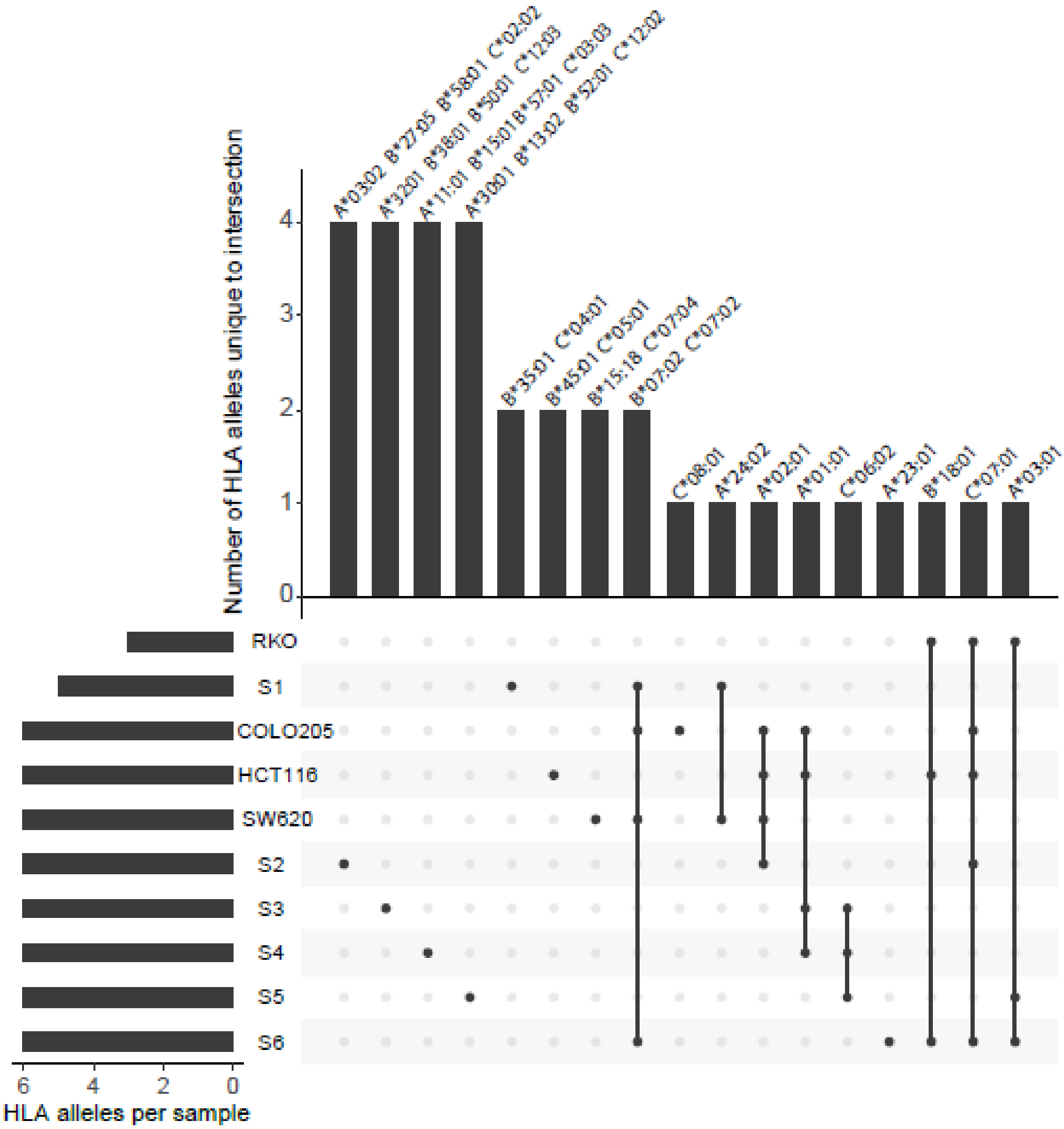

Supplementary figure S2. Upset plot of HLA alleles. UpsetR plot displaying the number of HLA alleles unique to a given intersection of samples, specifically MAPs that are unique to a given sample or that are uniquely shared by two samples.

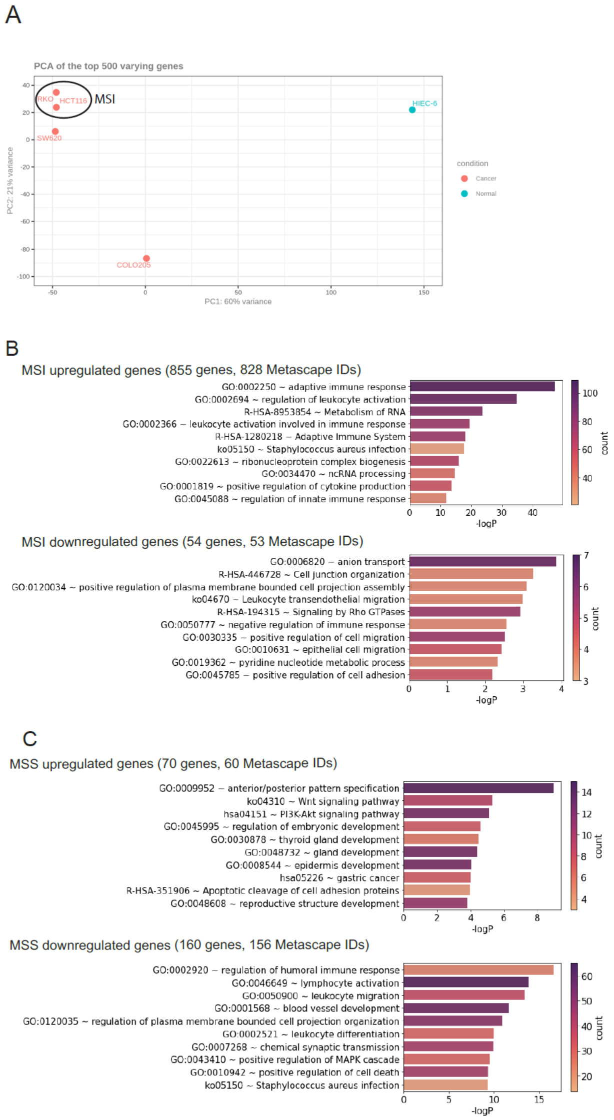

Supplementary figure S3. Transcriptomic profile of CRC-derived cell lines and GO term analysis of MSI and MSS primary tissue samples. A) Principal component analysis (PCA) of the top 500 varying genes of CRC-derived cell line and one normal intestinal cell line (HIEC-6) following paired-end RNA seq and gene read count normalization with DESeq2. Known MSI cell lines are encircled. B) GO term analysis of genes up/downregulated in MSI tumors compared to their adjacent NAT. Genes used for GO term analysis were those with $|log2FC| > 1$ when compared to their respective NAT using TPM normalized values and that were found to be uniquely differentially expressed in both of the MSI tumor samples (i.e. genes that were only up/downregulated in both of the MSI tumors but not any of the MSS tumors). C) GO term analysis of genes up/downregulated in MSS tumors compared to their NAT. Genes used for GO term analysis were those with $|log2FC| > 1$ when compared to their respective NAT using TPM normalized values and that were found to be uniquely differentially expressed in three or more of the MSI tumor samples (i.e. genes that were up/downregulated in at least three MSS tumors but neither of the MSI tumors).

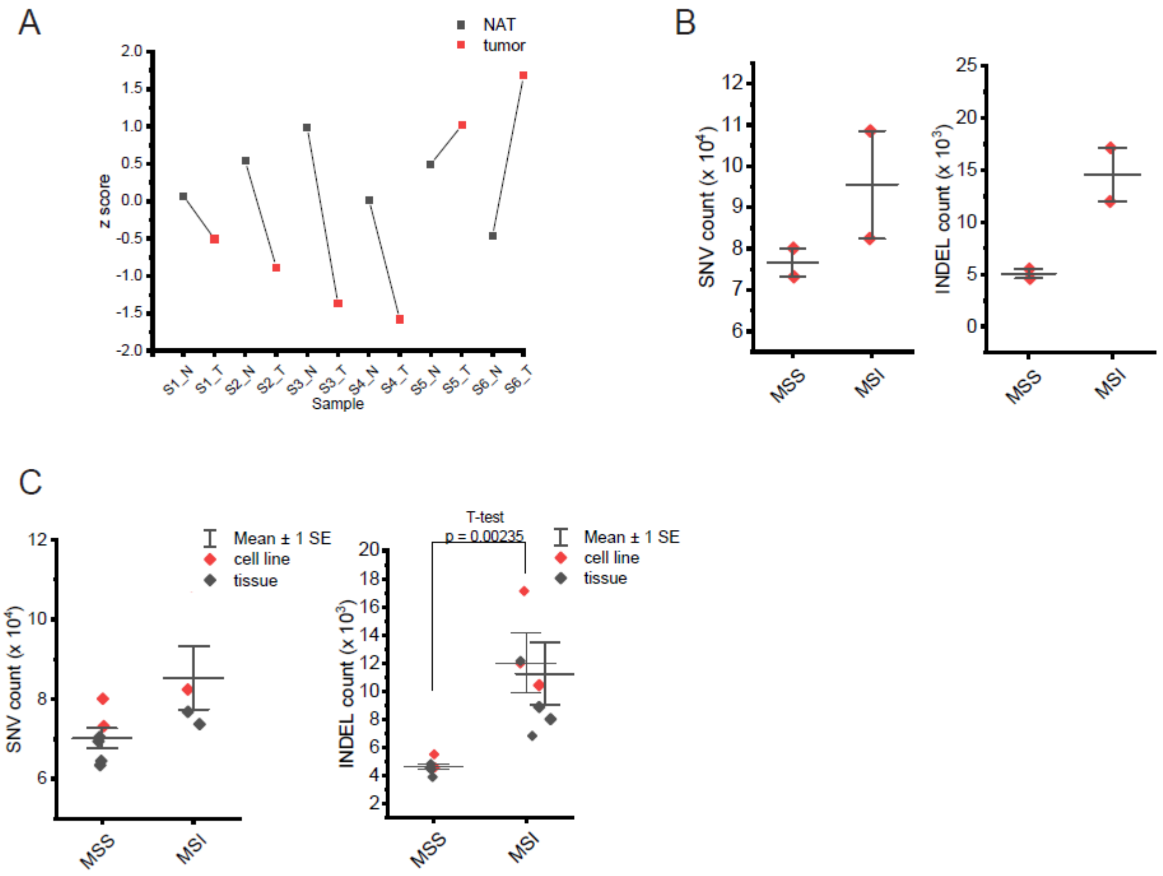

Supplementary figure S4. ssGSEA analysis of immune infiltration in CRC tissues and mutation profile of all samples. A) ssGSEA analysis of immune infiltration in tumor and matched NAT using genes described in Danaher et al. 2017 and GSVA R program (https://github.com/rcastelo/GSVA). B) Scatterplots displaying the SNV counts and INDEL counts of MSS and MSI CRC-derived cell lines determined by SNPEff genomic annotation, with mean and standard error bars. C) Scatterplots displaying the SNV counts and INDEL counts of all MSS and MSI samples (cell lines and tissues), determined by SNPEff genomic annotation, with mean and standard error bars. (SNV: p = 0.062; INDEL: p = 0.0024).

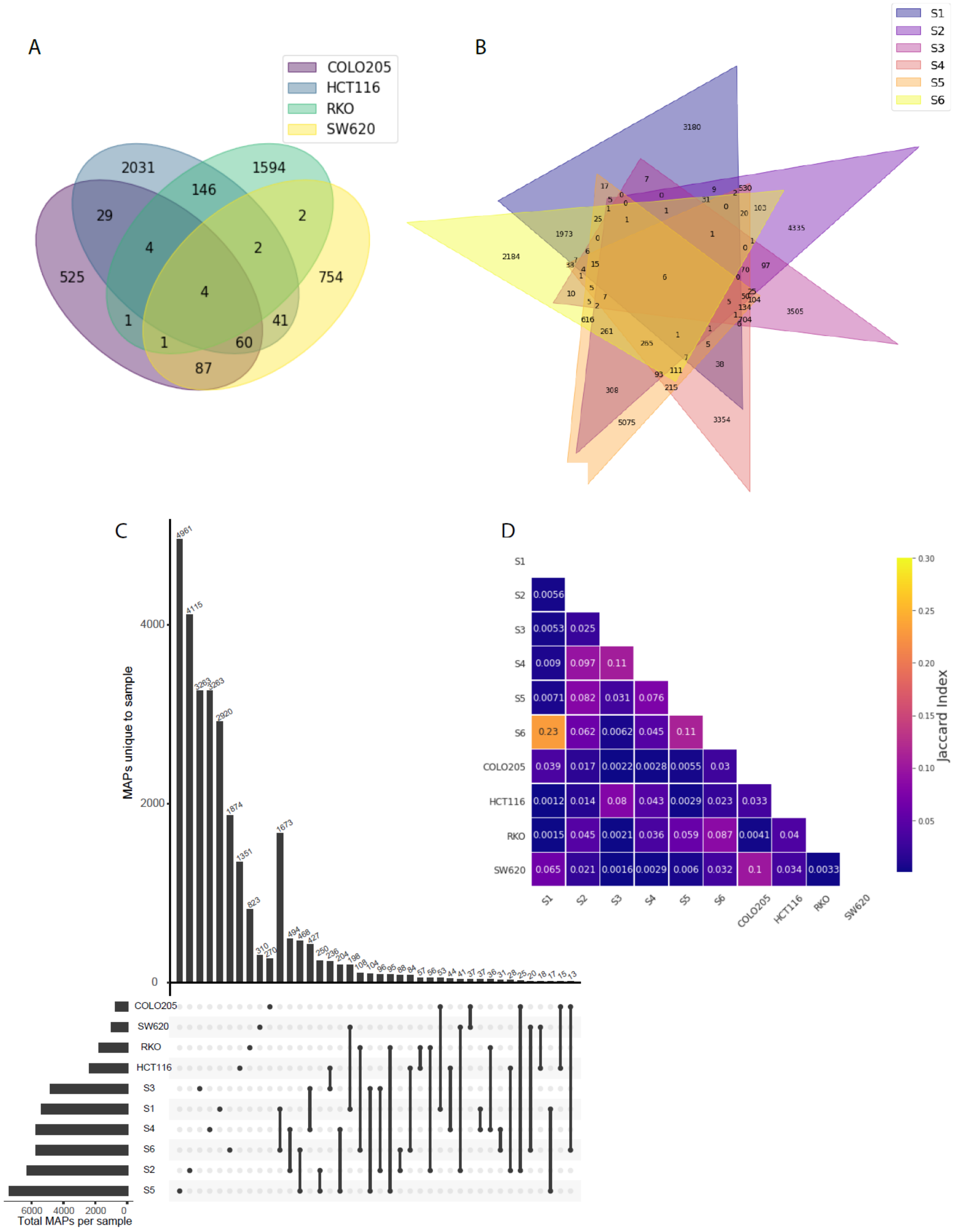

Supplementary figure S5. Overview of unique and shared MAPs in CRC-derived cell line and CRC/NAT tissue samples. A) Venn diagram displaying the overlap of MAPs in the MHC I immunopeptidomes of four CRC-derived cell lines. B) Venn diagram displaying the overlap of MAPs in the MHC I immunopeptidomes of six primary tissue samples. C) UpsetR plot displaying the number of MAPs unique to a given intersection of samples, specifically MAPs that are unique to a given sample or that are uniquely shared by two samples. D) Heatmap demonstrating the Jaccard index of MAP similarity between any two cell line or tissue samples.

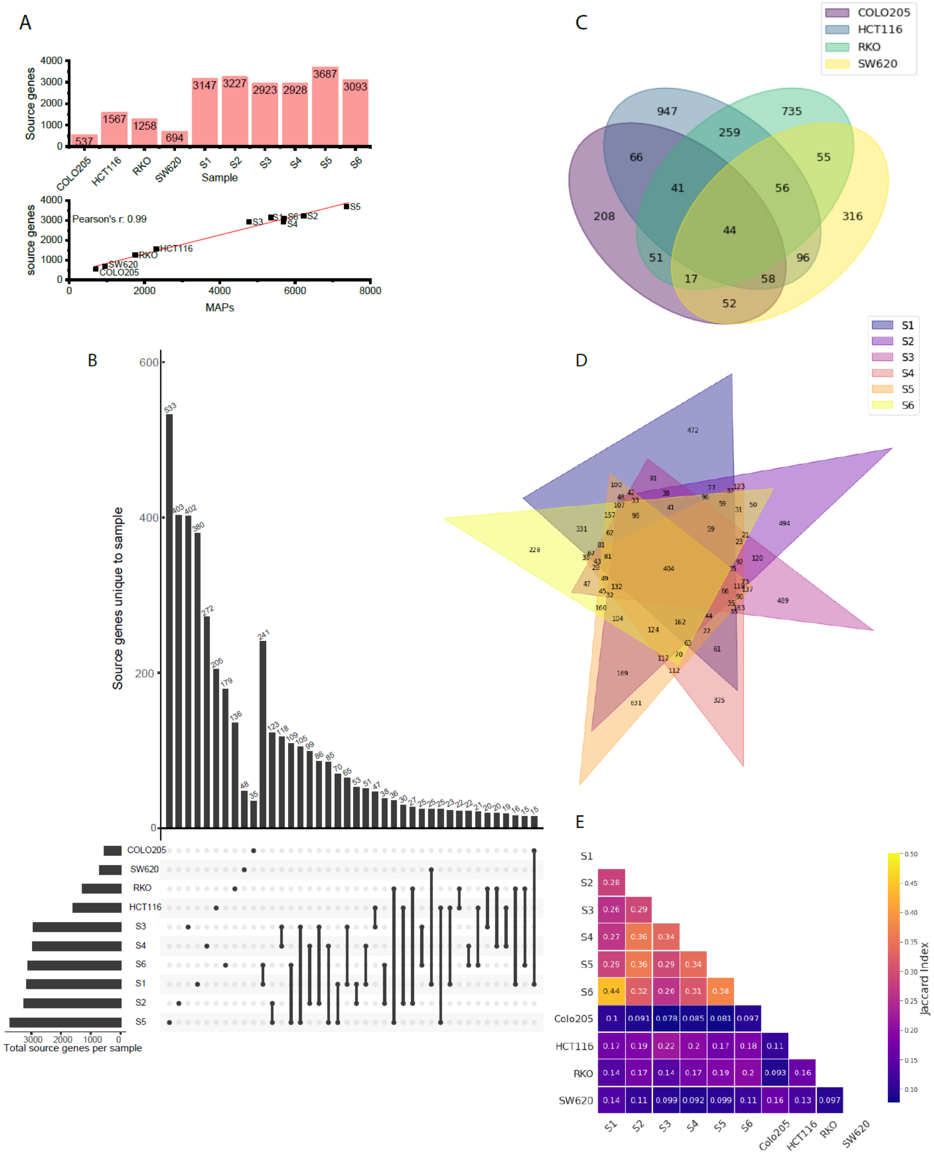

Supplementary figure S6. Overview of unique and shared MAP source genes in CRC-derived cell line and CRC/NAT tissue samples. A) Top panel: Bar chart displaying the number of unique source genes identified per sample. Bottom panel: Scatterplot indicating the correlation between the number of unique MAPs identified in each sample and the corresponding number of unique source genes (Pearson's r = 0.99). Source genes were identified for peptides from coding sequences; any peptide that mapped to more than one source gene was excluded. B) UpsetR plot displaying the number of source genes unique to a given sample or intersection, specifically source genes that are unique to a given sample or that are uniquely shared by two samples. C) Venn diagram displaying the overlap of source genes in the MHC I immunopeptidomes of four CRC-derived cell lines. D) Venn diagram displaying the overlap of source genes in the MHC I immunopeptidomes of six primary tissue samples. E) Heatmap demonstrating the Jaccard index of source gene similarity between any two cell line or tissue samples.

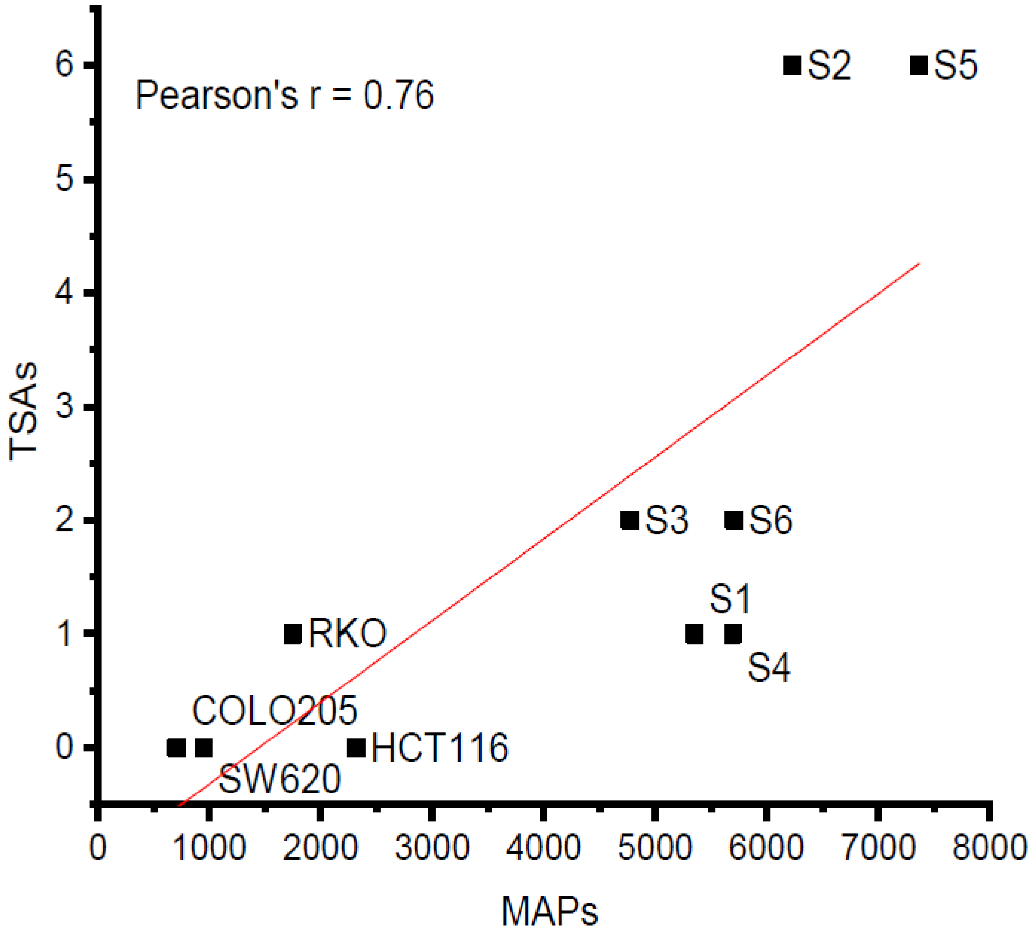

Supplementary figure S7. Correlation of MAPs and TSAs. Scatterplot indicating the correlation between the number of unique MAPs identified in each sample and the number of TSAs identified and validated (Pearson's r = 0.76).

A

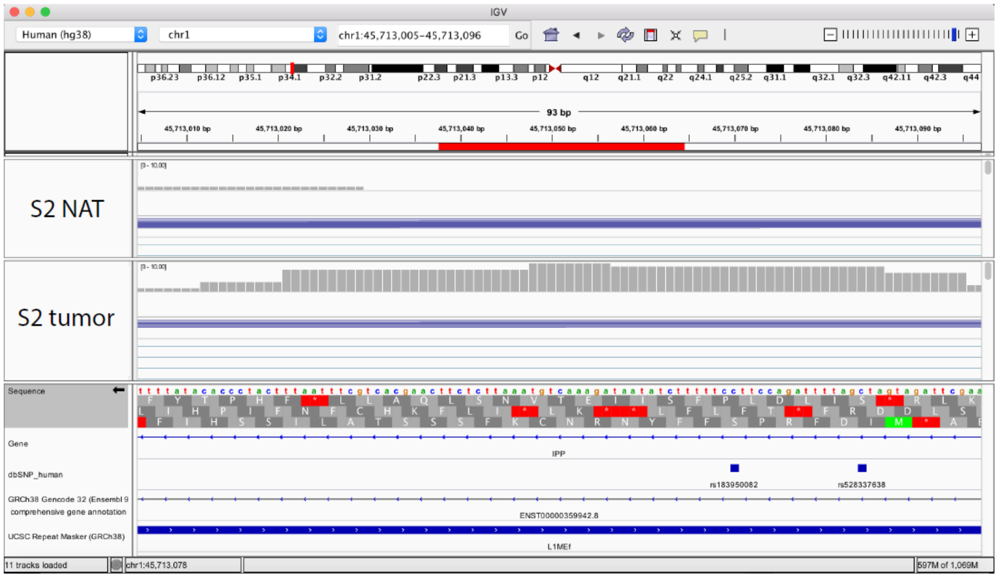

B

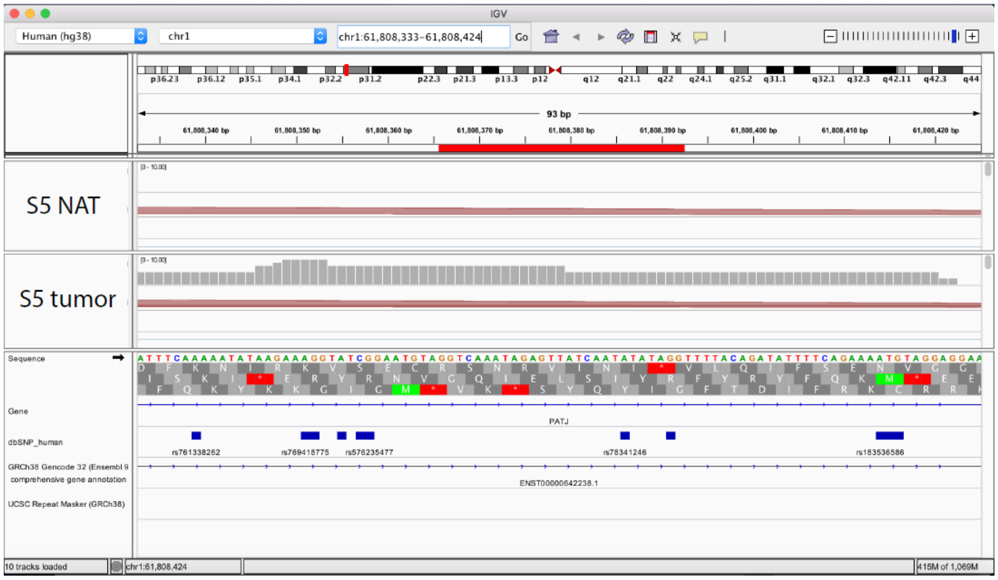

Supplementary figure S8. RNA expression of MCS in cancer and NAT. Interactive Genome Viewer screenshots of RNA-seq data for two peptides of interest, which act as a proof of concept that although the source transcript of a TSA or TAA may not be highly overexpressed in cancer compared to NAT, the MAP-coding sequence (MCS) can be significantly overexpressed in the tumor. A) MCS for aeTSA sequence SIIETVNSL in S2 RNA-seq data. The source transcript of SIIETVNSL has a log2FC of approximately -0.26 in CRC compared to NAT. B) MCS for aeTSA sequence GQIELSIYR in S5 RNA-seq data. The source transcript of GQIELSIYR has a log2FC of approximately 1.1 in CRC compared to NAT.

Supplementary figure S9 (1)

RMLLSHTGK / 4+

CRC_5peptides_5picomol_DDA_150621.raw [3357] (Synthetic – top)

RKO_TMT_10M_040920_1.raw [10154] (Endogenous – bottom)

m_{theo} = 1499.9012, m_{endo} = 1499.8997(−0.98 ppm), m_{syn} = 1499.9017(0.4 ppm)

r = 0.62, P < 1.66e−04, 95% CI 0.34 to 0.80

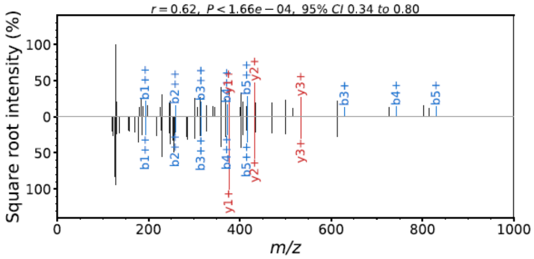

LPHRALSGI / 3+

CRC_1picomol_Synpept_incllist_CE40_100521.raw [19903] (Synthetic – top)

S1: CRC_026250T_TMT_200720_1.raw [10671] (Endogenous – bottom)

m_{theo} = 1191.7290, m_{endo} = 1191.7285(−0.43 ppm), m_{syn} = 1191.7288(−0.2 ppm)

r = 0.93, P < 8.19e−05, 95% CI 0.73 to 0.98

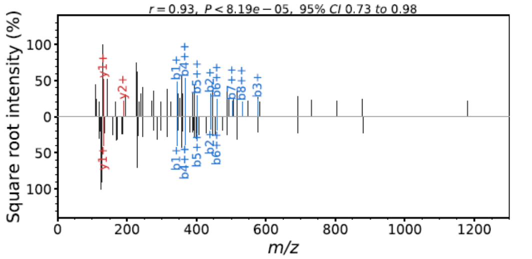

GTNPTAAVK / 2+

CRC_1picomol_Synpept_incllist_CE40_100521.raw [10270] (Synthetic – top)

S2: CRC_026241T_TMT_200720_1.raw [5837] (Endogenous – bottom)

m_{theo} = 1315.7864, m_{endo} = 1315.7864(0.0 ppm), m_{syn} = 1315.7870(0.4 ppm)

r = 0.99, P < 1.60e−15, 95% CI 0.98 to 1.00

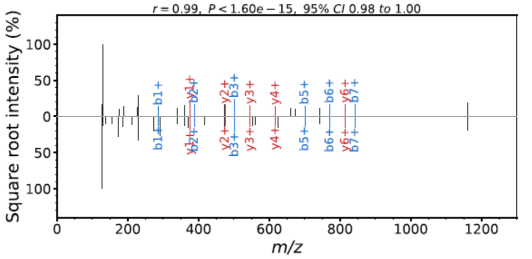

LRHKLVLNR / 4+

CRC_1picomol_Synpept_incllist_CE40_100521.raw [14050] (Synthetic – top)

S2: CRC_026241T_TMT_200720_1.raw [10093] (Endogenous – bottom)

m_{theo} = 1606.0560, m_{endo} = 1606.0529(−1.91 ppm), m_{syn} = 1606.0565(0.2 ppm)

r = 0.75, P < 1.44e−01, 95% CI −0.39 to 0.98

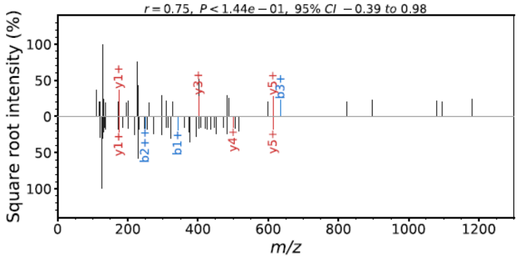

RIGGVGVEK / 3+

CRC_1picomol_Synpept_incllist_CE40_100521.raw [14247] (Synthetic – top)

S2: CRC_026241T_TMT_200720_1.raw [7890] (Endogenous – bottom)

m_{theo} = 1371.8604, m_{endo} = 1371.8596(−0.59 ppm), m_{syn} = 1371.8602(−0.1 ppm)

r = 0.99, P < 5.95e−20, 95% CI 0.97 to 0.99

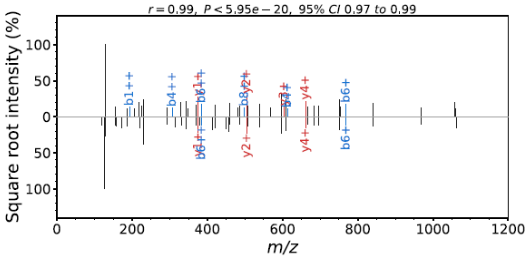

SIIETVNSL / 2+

CRC_1picomol_Synpept_incllist_CE40_100521.raw [31434] (Synthetic – top)

S2: CRC_026241T_TMT_200720_1.raw [15294] (Endogenous – bottom)

m_{theo} = 1203.6914, m_{endo} = 1203.6914(0.05 ppm), m_{syn} = 1203.6898(−1.3 ppm)

r = 0.91, P < 4.22e−03, 95% CI 0.51 to 0.99

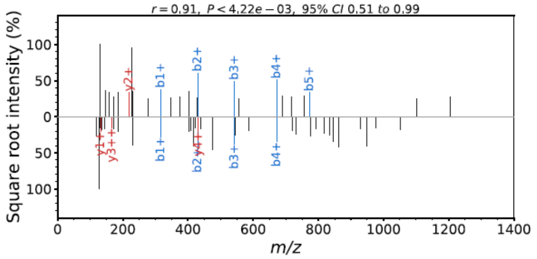

* TVNTQQYNTK / 2+

35CRCpep_TMT127_DDA_120721.raw [3290] (Synthetic – top)

S2: CRC_026241T_TMT_200720_1.raw [7245] (Endogenous – bottom)

m_{theo} = 1654.8932, m_{endo} = 1654.8826(−6.35 ppm), m_{syn} = 1654.8928(−0.2 ppm)

r = 0.61, P < 2.70e−03, 95% CI 0.25 to 0.82

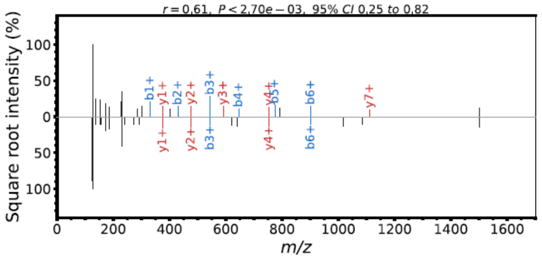

SVSHLHIFF / 3+

CRC_1picomol_Synpept_incllist_CE40_100521.raw [32222] (Synthetic – top)

S3: CRC_026214T_TMT_200720_1.raw [15049] (Endogenous – bottom)

m_{theo} = 1314.7288, m_{endo} = 1314.7288(0.0 ppm), m_{syn} = 1314.7270(−1.4 ppm)

r = 0.85, P < 3.13e−02, 95% CI 0.13 to 0.98

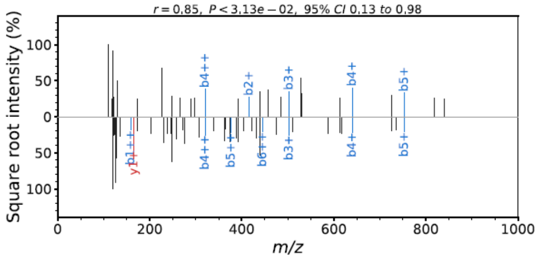

Supplementary figure S9 (2)

TTLENLPQK / 2+

CRC_1picomol_Synpept_incllist_CE36_100521.raw [17748] (Synthetic − top)

S4: *CRC_026075T_TMT_200720_1.raw [10017] (Endogenous − bottom)*

$m_{theo} = 1500.8916$, $m_{endo} = 1500.8922(0.4\,ppm)$, $m_{syn} = 1500.8918(0.1\,ppm)$

$r = 0.94$, $P < 1.71e-02$, 95% *CI* 0.34 to 1.00

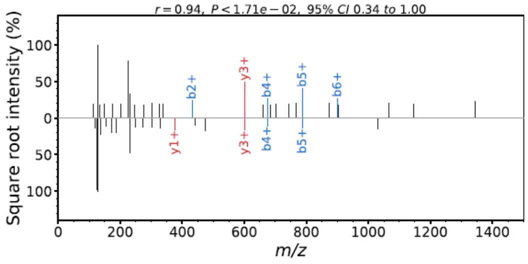

AQKLQVRI / 3+

35CRCpep_TMT127_incllist_120721.raw [8597] (Synthetic − top)

S5: *CRC_026240T_TMT_200720_1.raw [13155] (Endogenous − bottom)*

$m_{theo} = 1413.9073$, $m_{endo} = 1413.9055(-1.31\,ppm)$, $m_{syn} = 1413.9061(-0.8\,ppm)$

$r = 0.99$, $P < 6.36e-12$, 95% *CI* 0.97 to 1.00

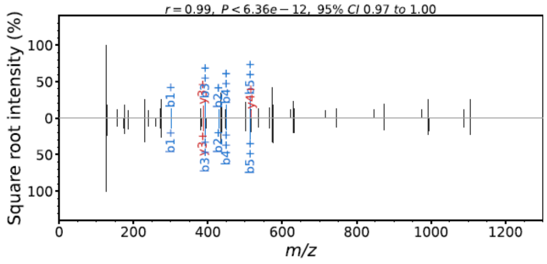

GQIELSIYR / 2+

CRC_1picomol_Synpept_incllist_CE40_100521.raw [35329] (Synthetic − top)

S5: *CRC_026240T_TMT_200720_1.raw [14678] (Endogenous − bottom)*

$m_{theo} = 1306.7447$, $m_{endo} = 1306.7440(-0.52\,ppm)$, $m_{syn} = 1306.7450(0.3\,ppm)$

$r = 0.69$, $P < 1.32e-02$, 95% *CI* 0.19 to 0.90

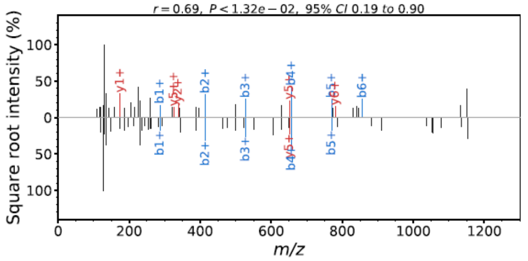

HGALSIRSI / 3+

CRC_1picomol_Synpept_incllist_CE36_100521.raw [21304] (Synthetic − top)

S5: *CRC_026240T_TMT_200720_1.raw [9370] (Endogenous − bottom)*

$m_{theo} = 1181.7084$, $m_{endo} = 1181.7079(-0.41\,ppm)$, $m_{syn} = 1181.7070(-1.1\,ppm)$

$r = 0.81$, $P < 1.57e-02$, 95% *CI* 0.23 to 0.96

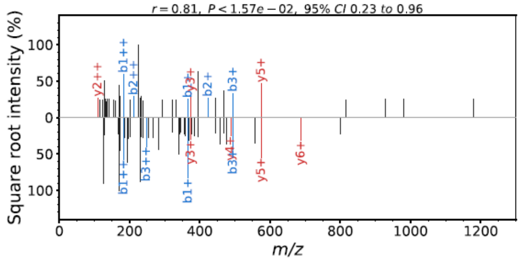

* RLMKFLPV / 3+

35CRCpep_TMT127_DDA_120721.raw [7273] (Synthetic − top)

S5: *CRC_026240T_TMT_200720_1.raw [19536] (Endogenous − bottom)*

$m_{theo} = 1460.9307$, $m_{endo} = 1460.9296(-0.74\,ppm)$, $m_{syn} = 1460.9311(0.2\,ppm)$

$r = 0.85$, $P < 1.91e-03$, 95% *CI* 0.47 to 0.96

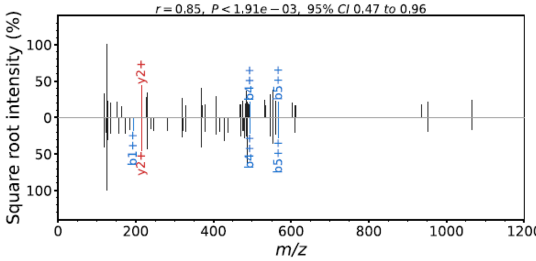

SLYISEERK / 3+

CRC_1picomol_Synpept_incllist_CE40_100521.raw [17137] (Synthetic − top)

S5: *CRC_026240T_TMT_200720_1.raw [13213] (Endogenous − bottom)*

$m_{theo} = 1581.9133$, $m_{endo} = 1581.9121(-0.76\,ppm)$, $m_{syn} = 1581.9130(-0.2\,ppm)$

$r = 0.86$, $P < 1.47e-03$, 95% *CI* 0.50 to 0.97

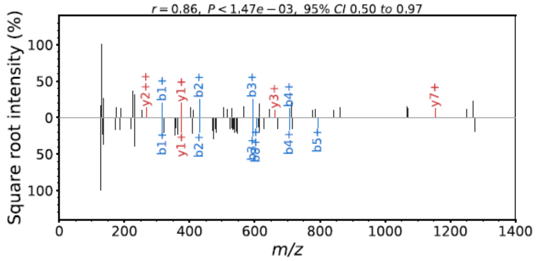

VQTAVLNV / 2+

CRC_5peptides_5picomol_DDA_150621.raw [5294] (Synthetic − top)

S5: *CRC_026240T_TMT_200720_1.raw [14178] (Endogenous − bottom)*

$m_{theo} = 1071.6490$, $m_{endo} = 1071.6488(-0.19\,ppm)$, $m_{syn} = 1071.6486(-0.4\,ppm)$

$r = 0.96$, $P < 3.06e-09$, 95% *CI* 0.89 to 0.99

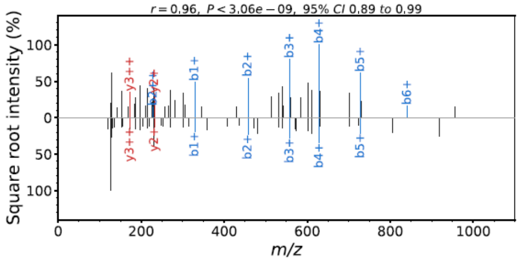

** RNRQVATAL / 3+

CRC_1picomol_Synpept_incllist_CE40_100521.raw [9743] (Synthetic − top)

CRC_026250T_TMT_200720_1.raw [4670] (Endogenous − bottom)

$m_{theo} = 1256.7516$, $m_{endo} = 1256.7508(-0.64\,ppm)$, $m_{syn} = 1256.7514(-0.1\,ppm)$

$r = 0.99$, $P < 2.06e-10$, 95% *CI* 0.97 to 1.00

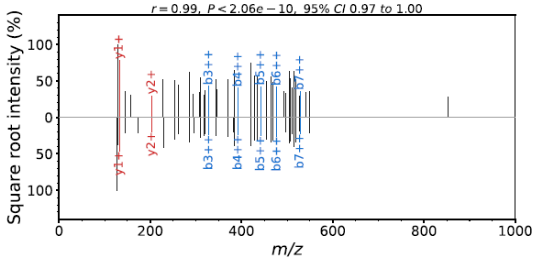

Supplementary figure S9 (3)

RNRQVATAL / 3+

CRC_1picomol_Synpept_incllist_CE40_100521.raw [9743] (*Synthetic − top*)

S6: *CRC_026019T_TMT_200720_1.raw* [4800] (*Endogenous − bottom*)

m_{theo} = 1256.7516, m_{endo} = 1256.7511(−0.4 ppm), m_{syn} = 1256.7514(−0.1 ppm)

r = 0.99, P < 7.45e−10, 95% *CI* 0.96 to 1.00

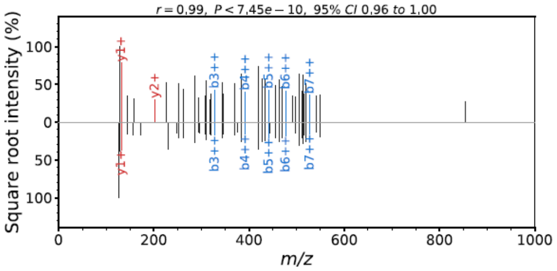

VEAPHLPSF / 2+

CRC_1picomol_Synpept_incllist_CE40_100521.raw [34361] (*Synthetic − top*)

S6: *CRC_026019T_TMT_200720_1.raw* [14903] (*Endogenous − bottom*)

m_{theo} = 1224.6704, m_{endo} = 1224.6706(0.16 ppm), m_{syn} = 1224.6690(−1.2 ppm)

r = 1.00, P < 6.49e−14, 95% *CI* 1.00 to 1.00

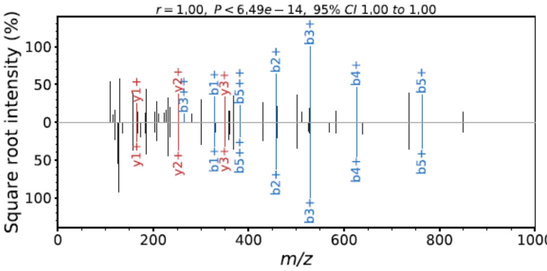

KIGEVIVTK / 3+

CRC_1picomol_Synpept_incllist_CE40_100521.raw [22989] (*Synthetic − top*)

S2: *CRC_026241T_TMT_200720_1.raw* [12444] (*Endogenous − bottom*)

m_{theo} = 1673.1061, m_{endo} = 1673.1064(0.18 ppm), m_{syn} = 1673.1043(−1.0 ppm)

r = 0.98, P < 4.26e−04, 95% *CI* 0.85 to 1.00

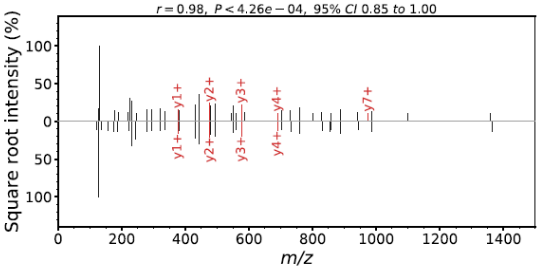

TRSTIILHL / 3+

CRC_1picomol_Synpept_incllist_CE40_100521.raw [32987] (*Synthetic − top*)

S3: *CRC_026214T_TMT_200720_1.raw* [12635] (*Endogenous − bottom*)

m_{theo} = 1281.7971, m_{endo} = 1281.7963(−0.65 ppm), m_{syn} = 1281.7960(−0.9 ppm)

r = 0.97, P < 2.69e−08, 95% *CI* 0.91 to 0.99

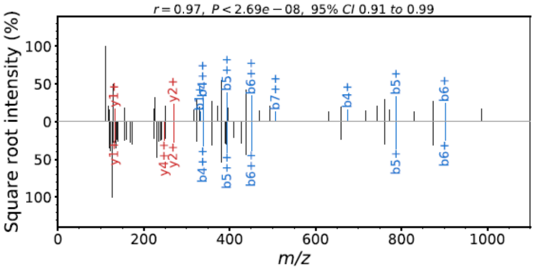

VLYRSVLLLK / 3+

CRC_1picomol_Synpept_incllist_CE36_100521.raw [38486] (*Synthetic − top*)

S6: *CRC_026019T_TMT_200720_1.raw* [12546] (*Endogenous − bottom*)

m_{theo} = 1661.1010, m_{endo} = 1661.1046(2.16 ppm), m_{syn} = 1661.1016(0.4 ppm)

r = 0.83, P < 5.12e−04, 95% *CI* 0.50 to 0.95

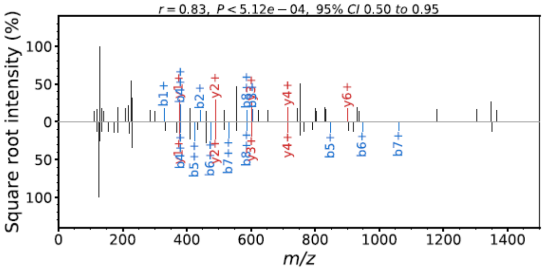

TYKYVDINTF / 2+

CRC_1picomol_Synpept_incllist_CE40_100521.raw [35408] (*Synthetic − top*)

S1: *CRC_026250T_TMT_200720_1.raw* [16029] (*Endogenous − bottom*)

m_{theo} = 1720.9440, m_{endo} = 1720.9454(0.81 ppm), m_{syn} = 1720.9446(0.3 ppm)

r = 0.99, P < 1.98e−08, 95% *CI* 0.96 to 1.00

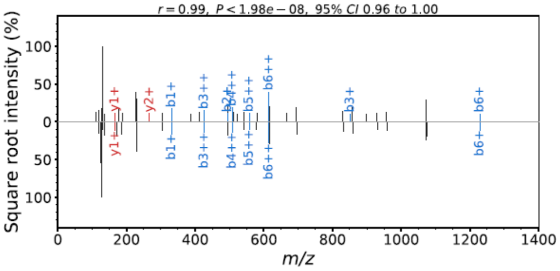

RYLEKFYGL / 3+

CRC_1picomol_Synpept_incllist_CE40_100521.raw [38099] (*Synthetic − top*)

S1: *CRC_026250T_TMT_200720_1.raw* [17307] (*Endogenous − bottom*)

m_{theo} = 1645.9597, m_{endo} = 1645.9585(−0.73 ppm), m_{syn} = 1645.9594(−0.2 ppm)

r = 0.95, P < 6.88e−05, 95% *CI* 0.79 to 0.99

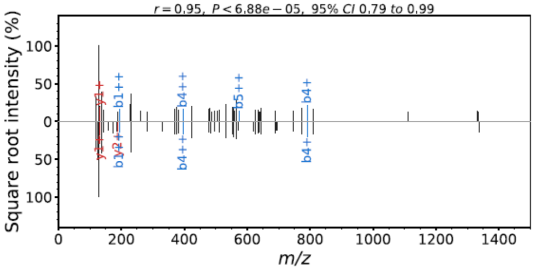

RYLEKFYGL / 3+

CRC_1picomol_Synpept_incllist_CE40_100521.raw [38423] (*Synthetic − top*)

S6: *CRC_026019T_TMT_200720_1.raw* [15960] (*Endogenous − bottom*)

m_{theo} = 1645.9597, m_{endo} = 1645.9594(−0.18 ppm), m_{syn} = 1645.9594(−0.2 ppm)

r = 0.98, P < 5.40e−06, 95% *CI* 0.89 to 1.00

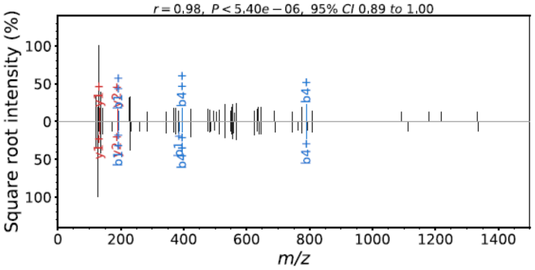

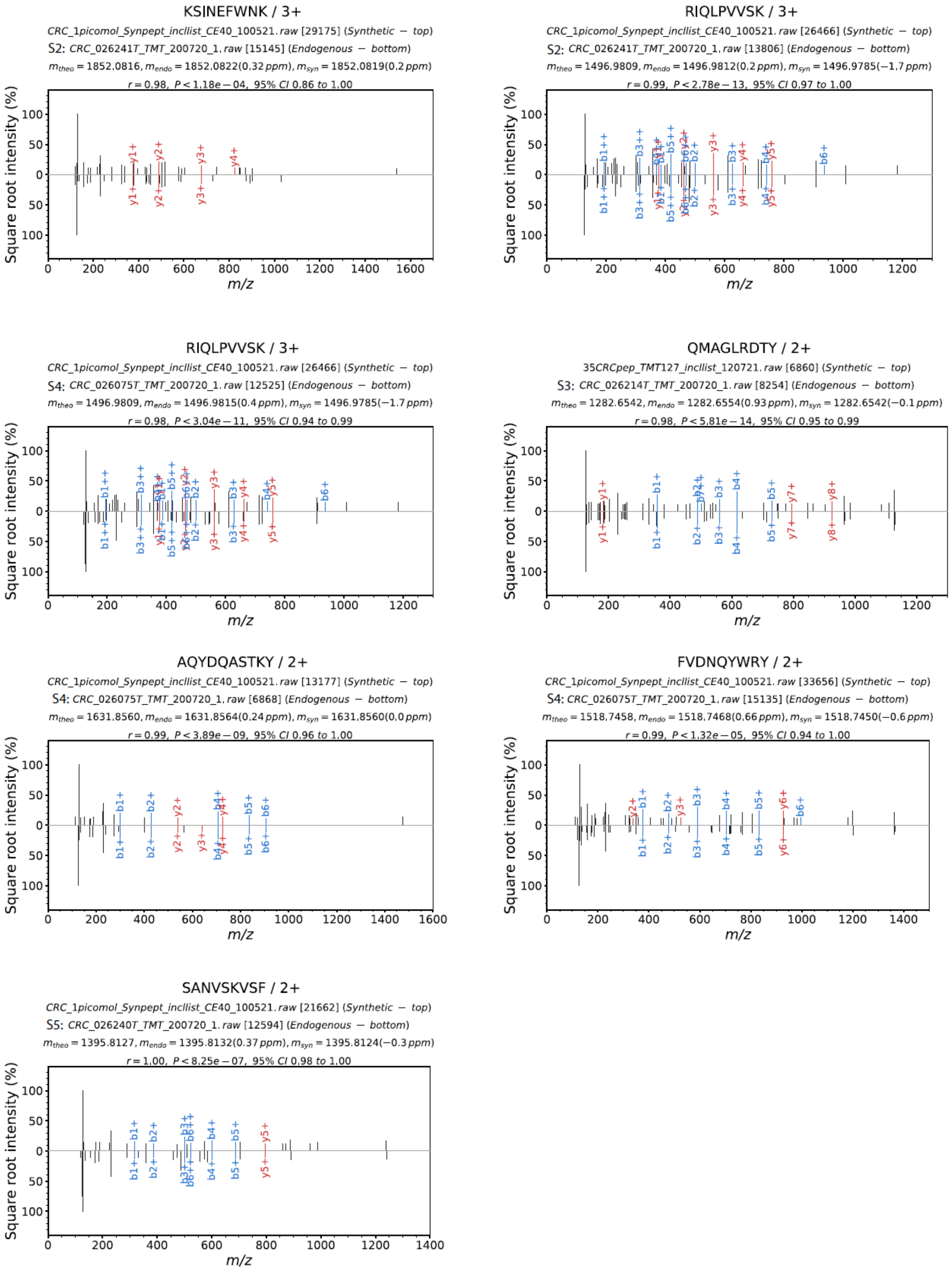

Supplementary figure S9. Mirror plots of TSA and TAAs. MS/MS correlations root scaled intensities of reproducibly detected peptide fragments of synthetic (top) and

endogenous (bottom) TSA and TAA sequences and charge states. b and y ions are displayed in blue and red, respectively. The text description, from top to bottom, includes: raw file name of synthetic peptide injection, followed by scan number in square brackets; raw file name of endogenous peptide injection, followed by scan number in square brackets; theoretical, endogenous, and synthetic masses listed as mtheo, mendo, and msyn, respectively, with mass errors are listed in brackets; Pearson correlation coefficient (r), p-value (P), and 95% confidence intervals (95% CI) for the correlation. Low intensity peptides (those with log10 median intensity of peptide fragments < 3) are indicated with *. The S1 mirror plot for RNRQVATAL sequence, which was identified as a TSA in S6 but also found to be overexpressed in S1, is indicated by **.

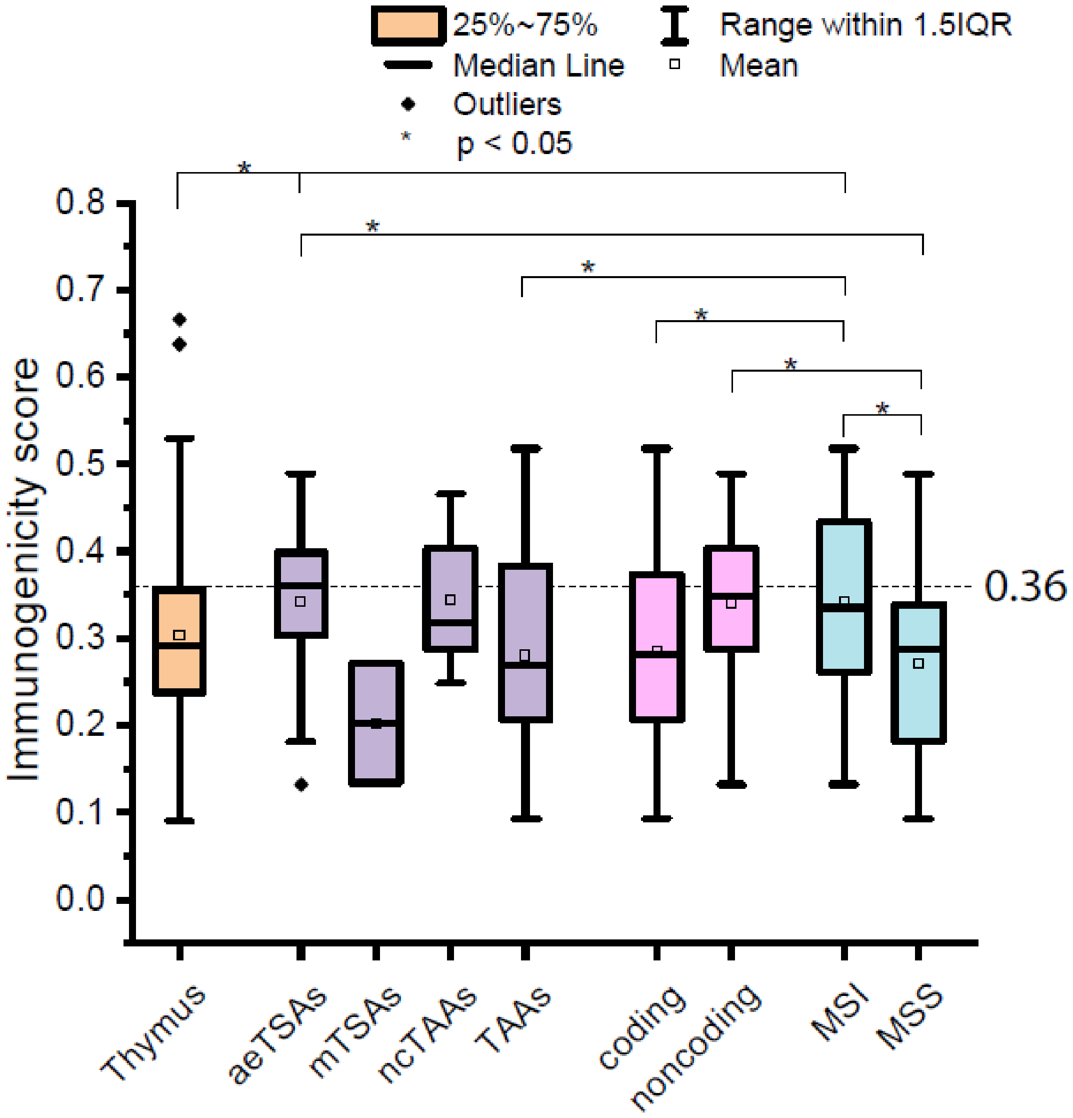

Supplementary figure S10. Immunogenicity of TSAs and TAAs. rEpitope immunogenicity scores of various groupings of validated TSAs and all TAAs compared to presumably non-immunogenic thymic peptides reported in Adamopoulou et al. 2013. rEpitope suggested threshold of immunogenicity for MHC I peptides (0.36) is indicated by the dashed line. This figure differs from main figure 6B in that it includes all TAAs reported in this work, not only the nine that were chosen for validation.
